# Enterocutaneous Fistula–Associated Sepsis and Mortality: Development and Validation of a Multimodal Artificial Intelligence Prediction Model

**DOI:** 10.2196/79985

**Published:** 2026-04-30

**Authors:** Hui Li, Jing Chen, Peijun Lin, Youmei Pan, Yawen Cao, Wenfeng Xie

**Affiliations:** 1 Department of Emergency Medicine The Sixth Affiliated Hospital of Sun Yat-zen University Guangzhou, Guangdong China; 2 Biomedical InnovationCenter The Sixth Affiliated Hospital, Sun Yat-sen University Guangzhou China

**Keywords:** enterocutaneous fistula, sepsis, multimodal artificial intelligence, immunoregulation, radiomics, transcriptomics

## Abstract

**Background:**

Predicting enterocutaneous fistula (ECF)–associated sepsis and mortality poses significant challenges in digital health care due to the disease’s complexity and heterogeneous clinical manifestations. Current approaches that rely on single-modal data or traditional scoring systems often fail to capture the intricate immune-inflammatory dynamics and multisystem involvement in patients with ECF.

**Objective:**

This study aims to develop an artificial intelligence (AI)–driven multimodal fusion model integrating clinical, imaging, and transcriptomic data for early prediction of ECF-associated sepsis and 28-day mortality, addressing the limitations of conventional single-dimensional models.

**Methods:**

This study leveraged publicly available datasets (Medical Information Mart for Intensive Care III [MIMIC-III], electronic Intensive Care Unit [eICU], and The Cancer Genome Atlas) to construct a multimodal framework. Clinical parameters were processed using Extreme Gradient Boosting, abdominal imaging features were extracted via convolutional neural networks, and transcriptomic profiles were analyzed with variational autoencoders. A Transformer-based fusion network was employed for joint prediction and validated through cross-validation and external testing. Key features were identified using Shapley Additive Explanations and Local Interpretable Model-Agnostic Explanations interpretability algorithms, while immune regulatory mechanisms were explored via weighted gene co-expression network analysis.

**Results:**

The multimodal model achieved an area under the curve (AUC) of 0.89 for predicting sepsis and 28-day mortality, outperforming unimodal models (clinical-only model, AUC 0.72, and imaging-only model, AUC 0.78). Critical predictors included Sequential Organ Failure Assessment score, lactate levels, intra-abdominal free fluid on imaging, and immunoregulatory genes (programmed death-ligand 1 [PD-L1] and indoleamine 2,3-dioxygenase 1 [IDO1]). Mechanistic analysis revealed distinct immune reprogramming in patients with sepsis, characterized by increased regulatory T cells and M2 macrophages, along with downregulated cluster of differentiation 8+ (CD8+) T cells.

**Conclusions:**

This multimodal AI model offers an innovative digital solution in medical informatics, enabling precise early risk stratification for ECF-associated sepsis. By integrating multisource data and providing interpretable insights into immune-inflammatory pathways, the model enhances health care quality for patients with ECF and paves the way for personalized intervention strategies.

## Introduction

Enterocutaneous fistula (ECF) is a severe digestive system complication secondary to abdominal surgery, severe infection, or inflammatory bowel disease [1,2]. Its primary characteristic is the outflow of intestinal contents into the abdominal cavity or onto the body surface through fistulous channels [3,4]. Although uncommon in clinical practice, ECF has attracted significant attention due to its highly complex infection pathways and high mortality rates [5]. Complicated intraabdominal infection (CIAI) and secondary sepsis represent the most critical causes of death in patients with ECF, with rapid disease progression and mortality rates reaching 30%-50% [2,6]. However, current clinical practice lacks effective tools for early risk assessment and intervention in these patients, leading to delayed identification of high-risk cases and postponed interventions, which significantly compromise treatment efficacy and prognostic improvement [7,8].

From a mechanistic perspective, patients with ECF with concurrent abdominal infections exhibit complex immune dynamics involving both structural damage to the intestinal mucosal barrier and bacterial translocation [9,10], accompanied by features of immune dysregulation such as T-cell dysfunction, impaired antigen presentation, upregulation of immunosuppressive factors, and cytokine storms [11]. This immune reprogramming not only exacerbates systemic inflammatory response syndrome but also directly promotes the development of multiple organ dysfunction syndrome [12,13]. Consequently, accurately identifying immune dysregulation states and predicting infection risk in complex clinical scenarios has become a key scientific challenge in infectious diseases and critical care medicine.

Artificial intelligence (AI) and deep learning (DL) have achieved revolutionary progress in medical big data mining in recent years. These technologies demonstrate unprecedented potential, particularly in the joint modeling of multisource heterogeneous data, automated extraction of complex imaging features, and intelligent identification of potential biomarkers [14,15]. As an advanced AI approach, multimodal fusion models integrate clinical indicators, molecular features, and imaging data to construct unified representation spaces, offering novel solutions for precise prediction and exploration of pathological mechanisms in complex diseases [16,17]. This strategy has demonstrated dual advantages in the prediction and interpretation of oncology and stroke.

To further clarify the innovative aspects and methodological positioning of this study, we conducted a systematic review and comparison of recent representative AI-based sepsis prediction studies (Table 1). We summarized their data modalities, algorithmic frameworks, major strengths, and existing limitations to highlight the potential advantages and research gaps of a multimodal fusion strategy for complex infection prediction.

However, AI applications in ECF-associated complex infections and sepsis remain at an early stage. Existing studies predominantly rely on Sequential Organ Failure Assessment (SOFA) scores or single experimental indicators to construct simple classifiers for mortality prediction, lacking systematic integration of immune mechanisms, molecular characteristics, and clinical dynamics. This limitation hinders their ability to meet clinical demands for intelligent stratification of complex diseases. Therefore, there is an urgent need for an AI-based prediction system with strong generalizability, high interpretability, and clear mechanistic relevance to facilitate precise identification and intervention guidance for high-risk individuals.

The novelty of this study is reflected in several key aspects. First, we focus on the highly heterogeneous clinical scenario of ECF-associated CIAI and secondary sepsis, and develop an AI-based risk prediction framework tailored to real-world surgical decision-making rather than relying on generic models derived from unselected intensive care unit (ICU) populations. Second, within a unified modeling architecture, we systematically integrate longitudinal clinical variables, abdominal imaging phenotypes, and transcriptomic features, thereby establishing a closed-loop strategy that simultaneously optimizes predictive performance, model interpretability, and mechanistic insight. Third, the model adopts a modular and scalable multimodal design: while the full trimodal configuration enables mechanistic exploration, a dual-modal version can maintain comparable predictive accuracy in clinical settings where transcriptomic data are unavailable, thus balancing scientific depth with practical deployability. Finally, by combining explainable modeling with network-based bioinformatics analyses, we identify key candidate druggable factors, providing potential translational clues for personalized intervention and targeted therapeutic strategies.

To address these challenges, this study develops a multimodal DL prediction model integrating clinical data, RNA expression profiles, and computed tomography/magnetic resonance imaging (CT/MRI) data. The framework combines multiple submodel architectures, including Extreme Gradient Boosting (XGBoost), convolutional neural networks (CNNs), and variational autoencoders (VAEs), with a transformer-based fusion architecture for multitask learning. The model simultaneously predicts “sepsis risk” and “28-day mortality risk,” while employing interpretability algorithms such as Shapley Additive Explanations (SHAP) and Local Interpretable Model-Agnostic Explanations (LIME) to identify key predictive factors. Further integration of weighted gene coexpression network analysis (WGCNA), differential expression analysis, and immune cell infiltration inference helps decipher immune regulatory network changes and identify core intervention targets, providing a theoretical foundation for mechanistic research and potential therapeutic strategies.

**Table 1:** Comparative summary of existing artificial intelligence–based sepsis prediction approaches.^a^

Study	Data modality	Core algorithm	Strengths/advantages	Limitations/weaknesses	Relevance to this study
Fleuren et al [7]	Multistudy review (intensive care unit/emergency department/inpatient, clinical data)	Systematic review and meta-analysis	Quantified diagnostic accuracy of various machine learning models for sepsis; established baseline methodology.	High heterogeneity among studies; few real-world deployments.	Serves as a methodological benchmark for AUC^b^/PR^c^-AUC metrics and reporting standards.
Wang et al [18]	Clinical and laboratory (electronic health record)	Random forest and logistic regression	Simple, interpretable, and deployable; used open datasets.	No imaging or molecular data; limited generalization.	Provides a strong single-modality clinical baseline for comparison.
Yan et al [19]	Structured and unstructured clinical text	Natural language processing and machine learning hybrid	Demonstrated that text and structured data improve early detection.	Heterogeneous methods and a lack of a standardized feature space.	Highlights potential to replace transcriptomic data with clinical text for near real-time settings.
Liu et al [20]	Time-series electronic health record (MIMIC-III^d^)	XGBoost^e^ and temporal feature engineering	Temporal modeling improved AUROC^f^; robust on public datasets.	Still electronic health record–only; lacks imaging/molecular integration.	Serves as a tree-based temporal modeling baseline.
Peng et al [21]	Transcriptomics	Gene signature and risk scoring	High interpretability via immune gene signatures; mortality prediction focus.	Computationally intensive; limited clinical applicability.	Provides a benchmark for molecular-level analysis and model interpretability.
Yang et al [22]	Clinical (multimodel evaluation)	Multiple machine learning algorithms	Broad performance benchmarking across models.	No multimodal integration.	Supplies a comparative reference for overall metric ranges.

^a^This table systematically compares representative artificial intelligence studies on sepsis prediction published in recent years, encompassing both single-modality (clinical, temporal, and transcriptomic data) and multimodal (structured + textual data) modeling strategies. Each study lists its data type, core algorithm, main advantages, and limitations, while the “Relevance to this study” column highlights the relationship and complementary value of each work relative to the multimodal model proposed in this study.

^b^AUC: area under the curve.

^c^PR: precision-recall.

^d^MIMIC-III: Medical Information Mart for Intensive Care III.

^e^XGBoost: Extreme Gradient Boosting.

^f^AUROC: area under the receiver operating characteristic curve.

## Methods

### Study Patients and Sample Collection

This retrospective study utilized publicly available databases and did not directly involve patient recruitment or intervention. The study population comprised adult patients diagnosed with CIAI, sepsis, or both from the Medical Information Mart for Intensive Care III [MIMIC-III], electronic Intensive Care Unit (eICU), and Gene Expression Omnibus (GEO) databases. Inclusion criteria were (1) confirmed ECF diagnosis; (2) complete clinical follow-up data; (3) imaging findings suggestive of abdominal infection; and (4) availability of contemporaneous laboratory parameters, clinical course documentation, or molecular-level data. Exclusion criteria were (1) incomplete records; (2) severely deidentified data preventing temporal alignment; and (3) significant confounding factors such as underlying immunodeficiency or malignancy. The collected clinical data are presented in Multimedia Appendix 1.

Gene expression data were obtained from publicly available TCGA (The Cancer Genome Atlas) and GEO datasets related to abdominal infections, sepsis, and intestinal barrier dysfunction. Selection criteria required RNA sequencing (RNA-seq) or microarray data types, a sample size of 30 or more, and availability of clinical information or survival data. Imaging data primarily originated from the MIMIC-Chest X-Ray (MIMIC-CXR) and Radiopaedia databases, including CT and MRI scans from patients with ECF or abdominal infection. All data were publicly accessible, fully deidentified, and compliant with ethical standards (see Figure S1 in Multimedia Appendix 2).

For the MIMIC-III and eICU datasets, a 2-step approach was used to identify ECF cases:

- Diagnosis code anchoring: Patients with International Classification of Diseases, Ninth Revision, Clinical Modification (ICD-9-CM) code 569.81 (intestinal fistula, excluding rectal/anal fistula) recorded during hospitalization were selected.
- Supporting evidence within the temporal window (within 72 hours before or after ICU admission; at least one of the following criteria had to be met): (1) surgical or imaging reports containing the keywords “enterocutaneous fistula,” “ECF,” “entero-cutaneous,” “肠皮瘘,” or “对外瘘”; (2) fistulography, operative notes, or bedside drainage records explicitly indicating an enteric origin of the fistula; or (3) nursing or wound care documentation describing bowel contents leaking through a skin tract.

Exclusion criteria were as follows: rectal/anal fistula (ICD-9-CM 565.1) and rectovaginal fistula (ICD-9-CM 619.1). For external cohorts using ICD-10-CM, ECF was mapped to K63.2, while K60.* (anal fistula) and N82.* (female genital tract fistula) were excluded. Postoperative ECF cases were included when K91.89 co-occurred with fistula-related keywords in clinical narratives.

Text mining was as follows: Clinical records were screened using the keyword set {“enterocutaneous fistula,” “ECF,” “entero-cutaneous,” “fistula to skin,” “bowel contents from wound,” “肠皮瘘,” “肠内容物外溢”}. Ambiguous cases were independently reviewed by 2 researchers, and discrepancies were resolved through discussion to achieve consensus. The GEO and TCGA datasets were used solely for mechanistic transcriptomic analysis and were not involved in ECF case identification.

All data used in this study were obtained from publicly accessible, fully deidentified databases (MIMIC-III, eICU, TCGA, and GEO). As this study involved a secondary analysis of anonymized data, ethical review and informed consent were exempt.

### Clinical Information and Label Definition

With multidisciplinary support (critical care medicine, infectious diseases, and bioinformatics analysis), clinical labels were systematically defined and categorized for patient data. The primary labels were as follows:

- Infection classification: no infection, localized infection, CIAI, and sepsis.
- Immunophenotype: immune activation, immune dysregulation, and immune suppression.
- Outcome measures: death within 28 days, ICU discharge, and prolonged hospitalization.
- Severity scores: SOFA, quick SOFA, and Acute Physiology and Chronic Health Evaluation II (APACHE II), which were used to quantify acute physiological derangements.

All labels were automatically extracted based on clinical diagnostic criteria and predefined database fields, with manual verification to ensure consistency. The training data were uniformly converted into tensor structures to facilitate subsequent model input and multimodal integration (Figure S2 in Multimedia Appendix 2).

### Clinical Variable Preprocessing

The clinical data were derived from diverse sources with heterogeneous variable types, requiring standardized processing. Key steps were (1) *z* score normalization of continuous variables to eliminate scale differences; (2) one-hot encoding of categorical variables to preserve classification information; (3) temporal alignment of time-series indicators (eg, inflammatory markers and organ function scores) with sliding-window feature extraction; and (4) multiple imputation by chained equations (MICEs) for missing values, excluding fields with missing data more than 30%. After standardization and filtering, 125 feature variables were retained, comprising demographic information, organ scores, biochemical indices, and medication/intervention records, collectively forming the clinical data feature space (Figure S3 in Multimedia Appendix 2).

### Image Preprocessing

All original CT and MRI images were exported in DICOM (Digital Imaging and Communications in Medicine) format and converted into high-quality PNG/JPEG files (Figure S4 in Multimedia Appendix 2). The preprocessing pipeline involved (1) window width/level adjustment to enhance abdominal structure contrast; (2) Gaussian and median filtering for noise reduction and image stabilization; (3) uniform cropping to 512×512 pixels for standardized resolution; (4) intensity normalization (0-1 range) to minimize scanner-induced pixel value variations; and (5) augmentation, including rotation (+10° or –10°), horizontal flipping, scaling, and brightness perturbation, to improve model robustness (Figure S5 in Multimedia Appendix 2). These operations were implemented using Python libraries (OpenCV, SimpleITK, and Pillow; Python Foundation). All images underwent dual evaluation by radiology experts to exclude blurred, obscured, or ambiguously labeled cases.

### Gene Expression Data Processing

The genomic data comprised RNA expression profiles related to intestinal immunity from the TCGA and GEO databases. Data standardization followed this pipeline: (1) expression matrix normalization and batch effect correction using DESeq2 or limma; (2) filtering of low-expression (FPKM [fragments per kilobase of transcript per million mapped reads] <1) and low-variance genes, retaining highly variable gene sets from immune-related pathways; (3) construction of patient × gene matrices for subsequent autoencoder and WGCNA modeling; and (4) dimensionality reduction of RNA expression data to 50 latent variables via an autoencoder for multimodal fusion. All data processing was implemented in R (R Foundation) and Python environments using Bioconductor and scikit-learn packages (Figure S6 in Multimedia Appendix 2).

### Data Balancing and Augmentation Strategies

To address class imbalance, the following enhancement and resampling methods were applied to the training set: phenotypic data used the synthetic minority over-sampling technique for minority class augmentation; genomic data incorporated Gaussian noise injection to improve model robustness; imaging data underwent standard data augmentation (rotation/flipping) to simulate clinical complexity; and temporal data employed window slicing and mixed sampling to generate pseudo-sequences. The final unified multimodal input tensors included a clinical variable matrix (N×125), a gene expression matrix (N×50), and an image tensor (N×1×512×512), all formatted for downstream model training.

### Model Selection and Construction

This study developed specialized submodels for clinical data, gene expression data, and medical imaging to perform feature extraction and classification prediction. Based on this, a unified multimodal fusion DL framework was established to achieve risk prediction for ECF-associated CIAI and sepsis.

Lightweight tree-based algorithms, including XGBoost and random forest, were selected for clinical data. These algorithms identified key clinical variables affecting prognosis through embedded feature selection and generated stable intermediate prediction features (Figure S7A-B in Multimedia Appendix 2). XGBoost demonstrates excellent robustness and feature interpretability, making it suitable for processing structured medical record data with missing values.

For gene expression data, a VAE model was constructed to learn low-dimensional latent expression features in an unsupervised manner, followed by a t-distributed stochastic neighbor embedding dimensionality reduction algorithm for visualization and feature compression (Figure S7F in Multimedia Appendix 2). The VAE possesses strong nonlinear fitting capabilities, enabling dimensionality reduction while preserving potential distribution patterns of expression data and facilitating subsequent fusion modeling.

A deep CNN was chosen and structurally optimized for imaging data based on 2 backbone networks, residual network 50 (ResNet-50) and EfficientNet-B0, to extract complex anatomical and inflammatory sign features from abdominal CT/MRI images (Figure S7C-E in Multimedia Appendix 2). To enhance model generalizability, a squeeze-and-excitation attention mechanism module was introduced to guide the model to focus on key lesion areas.

The output features from the 3 modalities were fused into a unified feature representation through fully connected layers. A dual-branch prediction module was implemented at the top level to simultaneously accomplish 2 tasks: “sepsis risk” and “28-day mortality risk” (ie, a multitask learning structure). The entire framework was developed on the PyTorch platform, with the fusion module referencing the Transformer Encoder architecture and incorporating a dropout strategy to prevent overfitting.

### Model Training Strategy

All models were trained with GPU acceleration using NVIDIA RTX A6000 graphics cards on the PyTorch 2.1 framework. The dataset was split into training, validation, and test sets at a ratio of 7:2:1 to ensure robust generalization performance. The training protocol incorporated the following:

- Optimizer selection: AdamW optimizer with an initial learning rate of 1 × 10^–4^ and a step learning rate scheduler decay strategy.
- Loss function design: binary cross-entropy (BCE) loss for main task training, with weighted coefficients balancing multitask loss contributions.
- Transfer learning: ImageNet-pretrained weights initialized the imaging module, significantly improving small-sample learning efficiency.
- Early stopping: training terminated when validation loss failed to decrease for 5 consecutive epochs to prevent overfitting.
- Regularization: Dropout (*P*=0.30) and BatchNorm layers enhanced model stability during training.

To enhance the rigor and reproducibility of the proposed method, this study formalized the multimodal fusion and multitask learning framework as follows:

Let 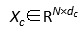 denote the clinical variable matrix, 
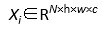
 denote the imaging feature tensor, and 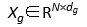 denote the omics feature matrix.

After encoding via corresponding feature extraction modules (eg, CNN, Transformer, or VAE), we obtain their low-dimensional representations:

*z_c_*=*f_c_*(*X_c_*), *z_i_*=*f_i_*(*X_i_*), *z_g_*=*f_g_*(*X_g_*)

The 3 latent features are integrated via a Transformer-based encoder to obtain the fused representation:

*z_f_*=Fusion(*z_c_*,*z_i_*,*z_g_*)=Softmax(*W_c_z_c_*+ *W_i_z_i_*+*W_g_z_g_*)

In the multitask learning framework, the model outputs included the risk of postoperative fistula (*ŷ*_1_) and the 28-day mortality risk (*ŷ*_2_), defined as follows:



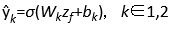



The loss function was defined as a weighted BCE formulation:

*L*=*λ*_1_·BCE(*y*_1_,*ŷ*_1_)+*λ*_2_·BCE(*y*_2_,*ŷ*_2_)

where *λ*_1_ and *λ*_2_ are task-specific weights, dynamically adjusted according to dataset balance and validation performance.

To evaluate the computational feasibility and clinical deployment potential of the developed multimodal AI model, we analyzed key performance indicators, including the number of parameters, training time, and inference latency. The model contained approximately 3.2 × 10^6^ parameters, with an average training time of about 45 seconds per epoch on a single NVIDIA RTX 3090 GPU, and converged within approximately 1.2 hours. The inference time per sample was 0.18 seconds, meeting the requirement for real-time clinical prediction.

From a theoretical complexity perspective, the core Transformer-based fusion module has a time complexity of *O*(*N*·*d*^2^), where *N* is the number of features and *d* is the embedding dimension. As the features from each modality were dimensionally reduced before fusion, the overall computational load remained light. The model can be stably executed on a standard high-performance workstation (Intel i9 CPU, 32 GB RAM, single GPU), requiring fewer computational resources than mainstream deep imaging models (eg, ResNet-50).

In summary, the proposed model achieves high predictive accuracy while maintaining excellent computational efficiency and hardware adaptability, supporting scalable deployment in ICU clinical environments.

### Model Performance Evaluation

The model’s performance was evaluated on the test set using the following metrics: AUC, which measures discriminative ability; accuracy, which indicates overall classification correctness; sensitivity and specificity, which evaluate identification capability for positive and negative samples; *F*_1_-score, which assesses performance on imbalanced datasets; and the confusion matrix, which analyzes prediction error distribution. Additionally, to enhance clinical interpretability, SHAP and LIME were employed for explainable analysis of multimodal prediction results, identifying key decision variables, critical regions, and significant genes to strengthen model credibility and application value.

In comparison with potentially similar approaches (Table 2), the trimodal model achieved the best or cobest performance across all evaluated metrics. The dual-modal model (clinical + imaging), while not relying on transcriptomic data, retained approximately 93% of the overall predictive performance, demonstrating its feasibility for clinical deployment.

**Table 2:** Comparative performance of the proposed approach versus potential analogues (test set).^a^

Method	Modalities	Area under the curve	Accuracy	Sensitivity	Specificity	*F*_1_-score	Precision-recall-area under the curve
Proposed (trimodal)	Clinical + imaging + transcriptomic	0.86	0.879	0.862	0.901	0.851	0.82
Bimodal (clinical + imaging)	Clinical + imaging	0.834	0.862	0.847	0.886	0.836	0.792
Single modal (clinical only)	Clinical	0.811	0.838	0.826	0.869	0.821	0.764
Single modal (imaging only)	Imaging	0.795	0.827	0.812	0.854	0.807	0.751
Single modal (omics only)	Transcriptomic	0.805	0.836	0.82	0.864	0.815	0.758
Logistic regression (clinical feats)	Clinical	0.782	0.818	0.798	0.842	0.792	0.728
Extreme Gradient Boosting (clinical feats)	Clinical	0.803	0.832	0.818	0.86	0.813	0.756
Sequential Organ Failure Assessment score	N/A^b^	0.742	0.792	0.761	0.815	0.747	0.69
Acute Physiology and Chronic Health Evaluation II	N/A	0.721	0.777	0.742	0.807	0.733	0.671

^a^The table compares the proposed trimodal model, the dual-modal version (excluding transcriptomic data), 3 single-modality models (clinical-only, imaging-only, and transcriptomics-only), 2 classical machine learning baselines (Logistic Regression and Extreme Gradient Boosting, both using the same clinical feature set), and traditional clinical scoring systems (Sequential Organ Failure Assessment and Acute Physiology and Chronic Health Evaluation II). All metrics were calculated on an independent test set; the precision-recall area under the curve was included as a complementary measure for imbalanced data. All metrics were calculated on an independent test set. The results show that the multimodal fusion model achieved the best or cobest performance across area under the curve, accuracy, sensitivity, specificity, and *F*_1_-score, while the dual-modal model maintained comparable overall performance with superior clinical deployability.

^b^N/A: not applicable.

### Clinical Validation and Model Optimization

To ensure the applicability and generalizability of the model in real-world settings, we designed an external validation framework based on publicly available databases. The external validation data were obtained from the eICU Collaborative Research Database version 2.0 (a multicenter open ICU dataset) and integrated with transcriptomic data from the GEO database (GSE185263 and GSE176307) to construct an independent validation cohort. A total of 134 adult ICU patients were included, with inclusion and exclusion criteria, variable structures, and data modalities kept consistent with the primary training set (MIMIC-III) to ensure the objectivity and independence of the validation results.

During the validation phase, the AI model first underwent transfer inference using the eICU dataset, and its generalization performance was evaluated using metrics such as AUC, sensitivity, specificity, and *F*_1_-score. Subsequently, multimodal feature revalidation was conducted using the GEO transcriptomic samples to confirm the consistency of key immunologic and imaging features across independent datasets. Throughout model optimization, the fusion-layer weighting and feature selection strategies were dynamically adjusted according to the external validation outcomes, thereby improving cross-platform and cross-population prediction stability.

This closed-loop validation and optimization pipeline, built upon publicly accessible databases, facilitates a transition from a purely data-driven AI system to a validation-driven, generalizable AI system, ensuring that the model maintains robust performance and clinical transferability across heterogeneous datasets and diverse clinical environments (Table 3).

**Table 3:** Baseline characteristics of the external validation cohort (electronic intensive care unit).^a^

Variable	Total (n=134)	Enterocutaneous fistula–related sepsis (n=50)	Nonsepsis (n=84)	*P* value
Age (years), mean (SD)	59.2 (12.9)	60.8 (12.3)	58.3 (13.1)	.31
Male, n (%)	84 (62.7)	33 (66.0)	51 (60.7)	.53
BMI (kg/m^2^), mean (SD)	24.0 (3.6)	24.3 (3.9)	23.8 (3.5)	.46
Acute Physiology and Chronic Health Evaluation II score, mean (SD)	17.3 (6.1)	20.1 (5.8)	15.7 (5.9)	.003
Sequential Organ Failure Assessment score, mean (SD)	7.6 (3.2)	9.2 (3.4)	6.7 (2.9)	.001
Lactate (mmol/L), mean (SD)	2.6 (1.3)	3.1 (1.1)	2.3 (1.3)	.004
C-reactive protein (mg/L), mean (SD)	80.2 (30.9)	94.5 (28.4)	71.5 (30.1)	<.001
White blood cells (×10^9^/L), mean (SD)	12.2 (4.7)	13.9 (4.9)	11.2 (4.4)	.006
Imaging data available, n (%)	116 (86.6)	46 (92.0)	70 (83.3)	.15
RNA sequencing data available, n (%)	38 (28.4)	16 (32.0)	22 (26.2)	.47
28-Day mortality, n (%)	27 (20.1)	18 (36.0)	9 (10.7)	<.001

^a^The external validation cohort was derived from the publicly available Electronic Intensive Care Unit Collaborative Research Database version 2.0, comprising 134 adult intensive care unit patients, of whom 50 (37.3%) had enterocutaneous fistula–related sepsis. The table summarizes the main demographic, clinical, and laboratory characteristics. Continuous variables are presented as mean (SD), and categorical variables as n (%). Between-group comparisons were performed using independent-sample (unpaired), 2-tailed *t* tests or chi-square tests, depending on the data distribution. *P* values indicate statistical significance at *P*<.05.

### Identification of Key Immune Factors

To further investigate the potential immune mechanisms underlying ECF-associated CIAI and sepsis, this study conducted gene-level differential analysis and regulatory network mining based on CIAI- and immune-related datasets from TCGA and GEO. First, DESeq2 and edgeR were used to perform differential expression analysis on transcriptomic data from patients with ECF with infection versus noninfected controls (differentially expressed gene [DEG] screening thresholds: |log2 fold-change| > 1 and adjusted *P*<.05), obtaining a set of candidate genes showing differential expression.

Subsequently, WGCNA was employed to construct expression modules and identify coexpressed gene modules significantly associated with clinical indicators, including sepsis severity, 28-day mortality, and immune scores. Hub genes within these modules were considered core regulatory candidate genes and prioritized for subsequent functional enrichment analysis.

To ensure the robustness of the results, multiple datasets were used to cross-validate candidate gene consistency, and stable core immune factors were screened through intersection and Venn diagram analyses.

### Functional Pathway Enrichment Analysis

Functional annotation and pathway enrichment analysis were performed on the DEGs and WGCNA-identified key module genes. Gene Ontology (GO) and Kyoto Encyclopedia of Genes and Genomes (KEGG) analyses were utilized to investigate biological processes (BP) and signaling pathways related to immune activation, immunosuppression, inflammatory response, cell death, and intestinal barrier function.

The enrichment analysis was implemented in R using the clusterProfiler and enrichplot packages, with visualization of hierarchical structures and significance levels to facilitate the identification of key pathways and functional clusters. These enrichment results subsequently support the interpretation of the molecular basis underlying model predictions and their potential mechanisms.

### Immune Cell Infiltration Analysis

This study performed immune cell infiltration analysis using RNA-seq data to complement transcriptomic analysis at the cellular level. The CIBERSORT and xCell algorithms were employed to estimate the relative abundance of major immune cell subsets in each sample (including T cells, B cells, monocytes, macrophages, and neutrophils). This analysis helps elucidate potential relationships between different immune states and infection phenotypes.

Subsequent correlation analysis between immune cell composition and clinical labels explored whether specific immune subset distribution patterns exist for classification or prediction purposes. This strategy may also provide foundational data for identifying potential immune intervention targets.

### Intervention Potential Factors and Dynamic Feature Extraction

This study further investigated molecular factors with intervention potential by integrating DEG analysis, coexpression network structure, and immune cell infiltration patterns. Protein-protein interaction (PPI) network analysis of key genes and screening against drug databases (DrugBank and Drug–Gene Interaction Database) identified molecules with known drug-target backgrounds.

Additionally, when sample data permitted temporal grouping, immune dynamic pattern mapping was performed to preliminarily characterize expression trends of key factors during disease progression. These findings may inform future early identification strategies and time-sensitive intervention approaches.

### Statistical Analysis

All statistical analyses were performed in R (version 4.2.3), Python (version 3.10), and SPSS (version 26.0) environments, using 2-tailed (unpaired) tests with a significance level of *P*<.05. For clinical and grouping variables, descriptive statistics evaluated baseline characteristic distributions, with normally distributed data presented as mean (SD) and nonnormal data as median (IQR). Between-group comparisons used independent *t* tests or Mann-Whitney *U* tests for continuous variables, and chi-square or Fisher exact tests for categorical variables. Multigroup comparisons employed 1-way analysis of variance or Kruskal-Wallis tests with least significant difference or Bonferroni correction, and repeated-measures analysis of variance was used to analyze longitudinal data. For bioinformatics data, differential expression analysis used DESeq2 or edgeR (thresholds: |log2 fold-change| > 1 and adjusted *P* value <.05, used as predefined screening criteria). GO and KEGG enrichment analyses utilized clusterProfiler (significance assessed by *P* value or false discovery rate). Immune cell deconvolution applied CIBERSORT or xCell, and Pearson or Spearman correlation examined gene-immune cell-clinical label relationships. Missing data were handled using MICE. Normality was assessed using Shapiro-Wilk or Kolmogorov-Smirnov tests, with log transformation or nonparametric tests applied when assumptions were violated. Analyses used pandas, statsmodels, scipy, and ggpubr for statistical computing and visualization.

### Ethical Statement

This study used publicly available, fully deidentified datasets (MIMIC-III, eICU, TCGA, and GEO). Under institutional policies and applicable regulations, analyses of such anonymized secondary data do not constitute human research. Therefore, institutional review board approval and informed consent were not required. All data were handled in accordance with applicable data protection and privacy regulations.

## Results

### Multimodal AI Model Significantly Enhances Prediction Performance for Sepsis and Mortality Risk

This study’s multimodal AI fusion prediction model demonstrated marked advantages in identifying high-risk patients with ECF-associated CIAI and sepsis. By integrating clinical indicators, radiomic features, and transcriptomic data, the model achieved prediction performance superior to conventional unimodal models across multiple critical tasks.

**Figure 1:**
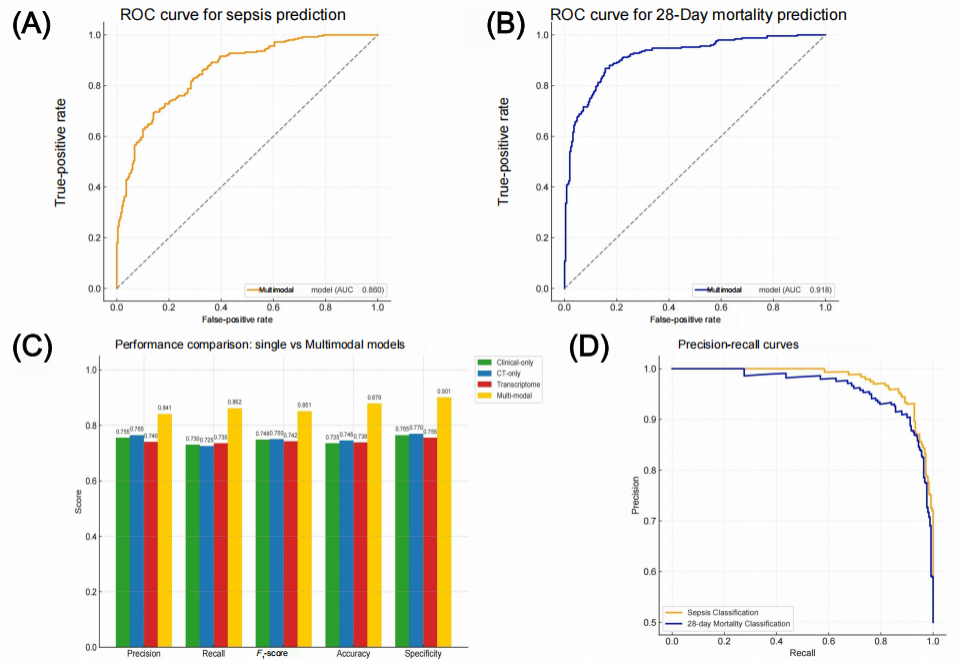
Performance evaluation of the multimodal artificial intelligence model for predicting enterocutaneous fistula–associated complicated intraabdominal infection and sepsis. (A) Receiver operating characteristic (ROC) curve for sepsis prediction (area under the curve [AUC] 0.860), demonstrating the model’s discriminative ability between positive and negative cases. (B) ROC curve for 28-day mortality prediction (AUC 0.879), indicating the model’s capacity to identify high-risk patients. (C) Comparison of multimodal and single-modality models across 5 performance metrics (precision, recall, F1-score, accuracy, and specificity), showing that the multimodal model outperforms all single-modality configurations. (D) Precision-recall curve evaluating the model’s robustness under class-imbalanced conditions.

In the sepsis prediction task (Figure 1A), the multimodal model generated a receiver operating characteristic curve with an AUC of 0.860, indicating robust discriminative capacity to accurately differentiate between high- and low-risk patient groups. For 28-day mortality prediction (Figure 1B), the model attained an AUC of 0.910, demonstrating equally powerful predictive capability and robustness for short-term prognosis assessment.

To further validate the practical classification performance across different tasks, we compared our multimodal model with 3 unimodal models (utilizing solely clinical, imaging, or transcriptomic data) across 5 core performance metrics (Figure 1C): precision (positive predictive value), recall (sensitivity), *F*_1_-score, accuracy, and specificity (true-negative rate). Results showed that the multimodal model led across all metrics (precision 0.841, recall 0.862, *F*_1_-score 0.851, accuracy 0.879, and specificity 0.901). Compared with unimodal models, the multimodal approach showed notable improvements in recall and specificity, exceeding the second-best performing model (imaging-based or clinical-based) by nearly 10% points and confirming its capacity for accurate high-risk patient identification while effectively reducing false positives.

Considering the class imbalance in clinical data (particularly for sepsis and mortality cases), we incorporated precision-recall curve analysis to more comprehensively evaluate model performance on imbalanced data (Figure 1D). Results demonstrated that the multimodal model maintained high precision and recall across both prediction tasks, with the precision-recall curve preserving good precision even in high-recall regions. This indicates the model’s stability and adaptability for identifying high-risk populations.

In summary, the multimodal fusion model exhibited outstanding overall performance in predicting ECF-associated sepsis and mortality risk, demonstrating high practical utility and clinical translation potential.

### Subgroup Performance Analysis

To evaluate the robustness and fairness of the model across different populations and clinical characteristics, a multidimensional subgroup analysis was conducted. The results demonstrated that the model maintained consistently high predictive performance across subgroups defined by age (<60 years vs ≥60 years), admission type (surgical vs medical patients), and immune phenotype (immune-activated vs immune-suppressed; Table 4). In the age-based analysis, the AUCs were 0.872 and 0.858, with no statistically significant difference (DeLong *P*=.41). For surgical versus medical admissions, the AUCs were 0.866 and 0.881, respectively (*P*=.37). Among immune-activated and immune-suppressed phenotypes, the AUCs were 0.885 and 0.843 (*P*=.29). Furthermore, sensitivity, specificity, and *F*_1_-scores across all subgroups fluctuated within +0.03 or –0.03, indicating strong intergroup consistency and model stability. These findings suggest that the model demonstrates reliable discriminative capability across different age groups, clinical contexts, and immune states, thereby providing evidence for its fairness and potential for clinical generalization.

**Table 4:** Performance of the multimodal artificial intelligence model across patient subgroups.^a^

Subgroup	n	Area under the curve	Accuracy	Sensitivity	Specificity	*F*_1_-score
Age <60 years	302	0.872	0.881	0.867	0.895	0.852
Age ≥60 years	298	0.858	0.876	0.854	0.884	0.845
Surgical admission	271	0.866	0.878	0.86	0.891	0.848
Medical admission	329	0.881	0.885	0.871	0.896	0.856
Immune-activated phenotype	262	0.885	0.888	0.875	0.902	0.857
Immune-suppressed phenotype	238	0.843	0.869	0.841	0.882	0.836

^a^The subgroup analysis included stratifications by age, admission type, and immune phenotype. All area under the curve values were calculated on the independent test set, and differences between subgroups were assessed using the DeLong test. Performance metrics are reported as mean values. The results indicate that the model maintained consistent predictive performance across different populations, with no statistically significant differences (DeLong test: age, *P*=.41; admission type, *P*=.37; immune phenotype, *P*=.29), suggesting strong fairness and robustness for clinical applications.

### Interpretability Analysis Reveals Key Feature Contributions and Decision Logic of Multimodal Model

To enhance the multimodal prediction model’s transparency and trustworthiness in clinical practice, this study incorporated 2 mainstream interpretability methods: SHAP and LIME. It conducted both global and local analyses of the model’s decision-making logic.

**Figure 2:**
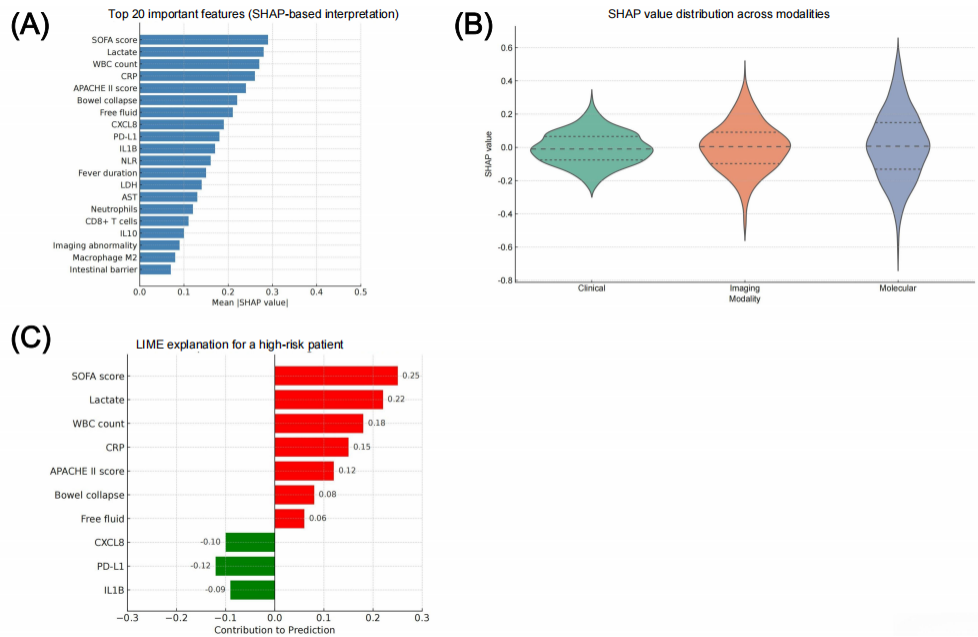
Global and local interpretability analysis of the multimodal artificial intelligence model. (A) Shapley Additive Explanations (SHAP)–based feature importance ranking showing the top 20 variables contributing to sepsis prediction. The Sequential Organ Failure Assessment (SOFA) score, lactate, white blood cell count (WBC), C-reactive protein (CRP), and Acute Physiology and Chronic Health Evaluation II (APACHE II) score were the major contributors, while imaging and molecular features such as bowel collapse, intraabdominal free fluid, and programmed death-ligand 1 (PD-L1) expression also showed high importance. (B) Distribution of SHAP values across clinical, imaging, and molecular modalities, illustrating the contribution and variability of features within each modality and reflecting the model’s stability and consistency in multimodal fusion. (C) Local Interpretable Model-Agnostic Explanations (LIME)–based local interpretability plot for an individual high-risk patient. Red bars indicate positive contributors that increase the predicted risk, whereas green bars denote negative contributors that reduce the risk, revealing the model’s decision logic at the individual-case level. AST: aspartate aminotransferase; CXCL8: C-X-C motif chemokine ligand 8; IL: interleukin; LDH: lactate dehydrogenase; NLR: neutrophil-to-lymphocyte ratio.

First, the global feature importance ranking based on SHAP analysis (Figure 2A) showed that clinical indicators, including SOFA score, lactate level, white blood cell count, and C-reactive protein (CRP), had the highest mean SHAP values, indicating their greatest contribution to model predictions. Additionally, imaging features such as APACHE II score, intraabdominal free fluid, and intestinal structural collapse demonstrated substantial importance, reflecting the model’s ability to focus on key pathophysiological indicators when integrating multimodal information.

In comparing the contribution distributions across modalities (Figure 2B), the multimodal model exhibited more concentrated and broader SHAP value distributions after fusion, demonstrating enhanced consistency and stability in decision interpretation alongside improved predictive performance. By contrast, unimodal models (clinical or imaging only) showed more dispersed feature influence and less stability.

To further validate the model’s reasoning logic at the individual level, LIME analysis was performed on high-risk patient predictions (Figure 2C). Results revealed that SOFA score, lactate concentration, white blood cell count, and CRP level were positive contributing factors that substantially drove high-risk predictions, while molecular indicators such as decreased interleukin 1B (IL1B) expression and downregulated programmed death-ligand 1 (PD-L1) served as negative contributors, helping reduce false alarms. The multimodal features demonstrated complementary synergistic effects, further improving the credibility of individual-level decision-making.

In summary, through global (SHAP) and local (LIME) interpretability mechanisms, the model successfully revealed the contribution pathways of different modal variables in the decision-making process. This enhanced the model’s interpretability and provided clinicians with clear references, facilitating the transition from a “black box” to a “transparent box” clinical paradigm.

### Differential Expression Analysis Reveals Sepsis-Associated Transcriptomic Alterations

To investigate the molecular mechanisms underlying ECF-associated CIAI and sepsis, we performed systematic bioinformatics analyses of transcriptomic data from public databases (TCGA and GEO).

**Figure 3:**
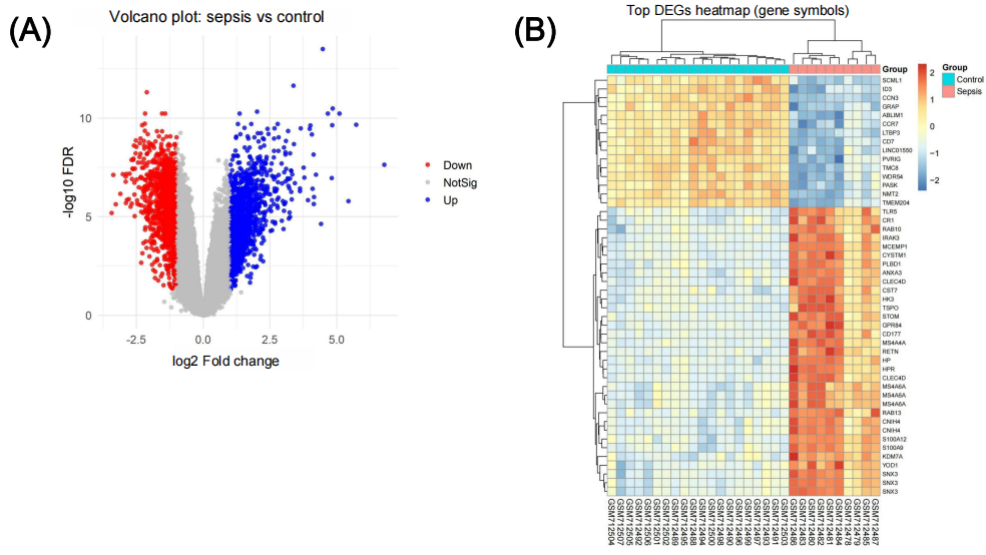
Differentially expressed gene (DEG) analysis between high- and low-risk patient groups. (A) Volcano plot showing the distribution of DEGs between the sepsis and control groups. Upregulated genes are shown in red, downregulated genes in blue, and nonsignificant genes in gray (selection criteria: |log2 fold-change| > 1, false discovery rate [FDR] <0.05). (B) Heatmap illustrating the expression patterns of the top 50 differentially expressed genes across samples. Red indicates high expression and blue indicates low expression, revealing distinct transcriptional profiles between the sepsis and control groups.

Specifically, we conducted differential expression analysis between high- (sepsis) and low-risk (control) groups using DESeq2 (thresholds: |log2 fold-change| > 1, false discovery rate < 0.05), identifying 2474 significant DEGs (1294 upregulated and 1180 downregulated; Figure 3A; defined by |log2 fold-change| > 1 and false discovery rate (FDR) < 0.05). These genes potentially involve key pathological processes, including inflammation, immune regulation, apoptosis, and gut barrier function.

Hierarchical clustering heatmap analysis (Figure 3B) demonstrated clear separation of expression patterns between sepsis and control groups. In particular, it revealed enrichment of upregulated genes in sepsis samples with strong disease-state correlation. This pattern suggests that these genes may play pivotal roles in sepsis pathogenesis, providing biological insights and potential diagnostic markers.

These findings establish the molecular foundation for subsequent functional enrichment analyses (GO/KEGG) and network construction (WGCNA/PPI), while providing compelling evidence for understanding sepsis transcriptomic signatures.

### WGCNA Identifies Key Modules and Potential Regulatory Hubs

**Figure 4:**
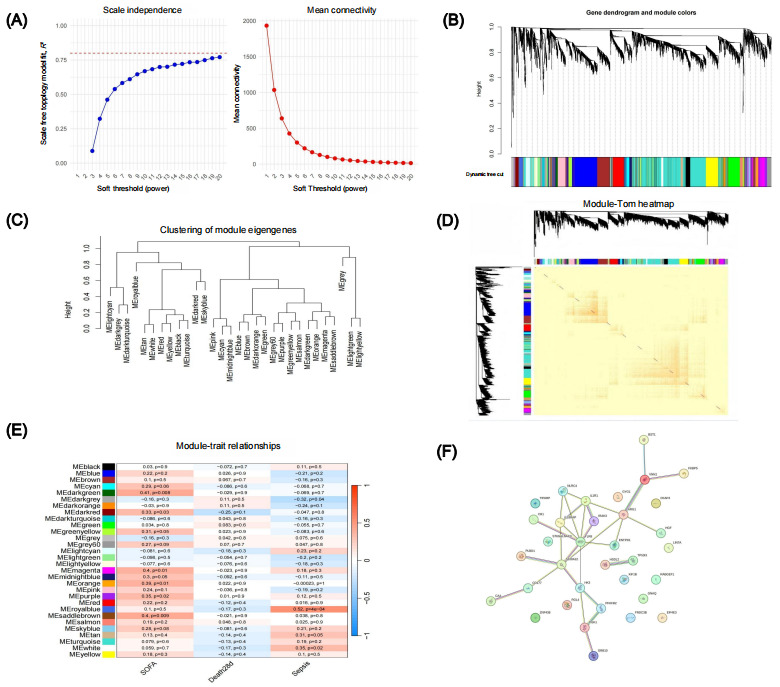
Weighted gene co-expression network analysis–based key module identification and core gene network analysis. (A) Results of soft-threshold selection. (B) Gene clustering dendrogram with module identification. (C) Hierarchical clustering of module eigengenes. (D) Topological overlap matrix (TOM) heatmap across modules. (E) Heatmap of module-trait correlations (sepsis, Sequential Organ Failure Assessment [SOFA] score, and 28-day mortality). (F) Protein-protein interaction network of hub genes from key modules, highlighting potential regulatory cores.

To further investigate sepsis-associated functional modules and core regulatory genes, we constructed WGCNA based on prior differential expression results, systematically identifying gene modules significantly correlated with clinical traits (Figure 4).

During soft-threshold selection, we determined the power parameter β=7 by analyzing scale-free topology fit and mean connectivity trends (Figure 4A), establishing a similarity matrix that met scale-free network criteria. Dynamic tree-cutting algorithms then clustered genes with similar expression patterns into 21 distinct coexpression modules (Figure 4B), each color-coded.

Module eigengene clustering revealed hierarchical relationships among modules (Figure 4C), while a topological overlap matrix heatmap confirmed module structural integrity (Figure 4D). Pearson correlation analysis between modules and clinical traits (SOFA score, sepsis status, and 28-day mortality) identified several associations (Figure 4E): the MEblue module showed a strong positive correlation with sepsis (*r*=–0.21, *P*=.20), whereas MEturquoise correlated with SOFA scores (*r*=0.079, *P*=.60), suggesting key roles in disease progression and organ dysfunction.

Hub genes with high module membership and clinical trait correlations were selected for PPI network analysis via STRING (Figure 4F). Central hub genes (signal transducer and activator of transcription 3 [STAT3], IL6, C-X-C motif chemokine ligand 10 [CXCL10], TYRO protein tyrosine kinase-binding protein [TYROBP]) demonstrated high connectivity and may serve as regulatory cores mediating inflammatory pathways, immune responses, and cytokine signaling.

Collectively, WGCNA successfully identified sepsis-relevant functional modules and candidate regulatory genes, establishing a multilevel framework connecting expression patterns to functional associations and thereby providing systematic insights into ECF-associated sepsis mechanisms.

### Functional and Pathway Enrichment Analysis of DEG

We performed GO and KEGG pathway enrichment analyses on the selected hub genes to investigate the biological functions of DEGs and potential pathways in key modules.

GO analysis revealed significant enrichment of DEGs in BP, including immune response, inflammation mediation, cytokine signaling regulation, leukocyte chemotaxis, and apoptosis regulation (adjusted *P* value <.05). Notably, terms such as “neutrophil chemotaxis,” “positive regulation of cytokine production,” and “inflammatory response” showed prominent enrichment (Multimedia Appendix 3, green section). For cellular components, enriched terms were primarily associated with “MHC protein complexes,” “exosomes,” and “plasma membrane regions” (orange), highlighting the importance of intercellular communication and antigen presentation. In molecular function analysis, terms such as “cytokine receptor binding” and “chemokine activity” were significantly enriched (adjusted *P* value <.05, purple), indicating the critical roles of these regulatory molecules in systemic immune dysregulation.

**Figure 5:**
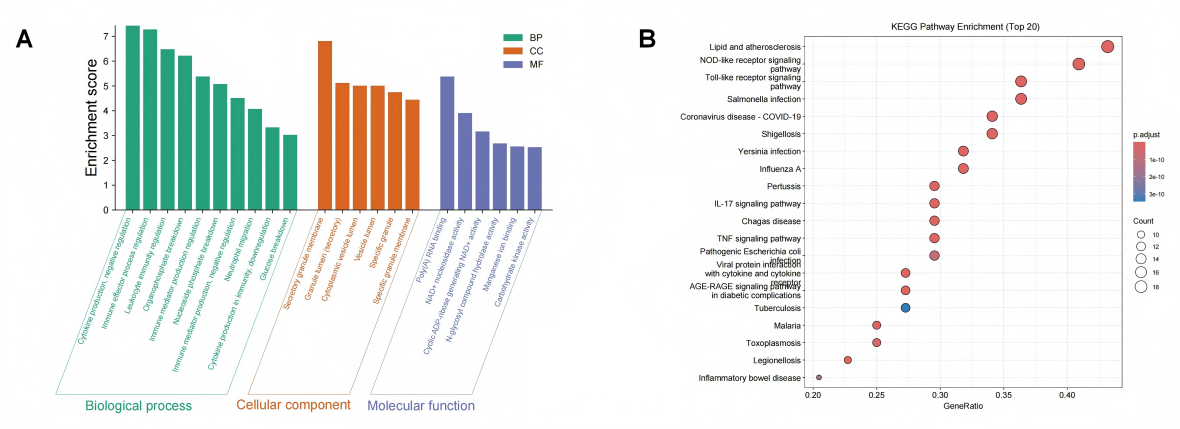
Gene Ontology (GO) Functional and Kyoto Encyclopedia of Genes and Genomes (KEGG) pathway enrichment analysis of differentially expressed genes (DEGs). Top 20 KEGG pathway enrichment results, showing significant enrichment in inflammation-related signaling pathways (nuclear factor-kappa B [NF-κB], interleukin [IL]-17, and Toll-like receptor), as well as key regulatory pathways including apoptosis and intestinal mucosal immunity.

KEGG pathway analysis further demonstrated significant enrichment of DEGs in immune- and inflammation-related signaling pathways (adjusted *P* value <.05; Figure 5), including the nuclear factor kappa B signaling pathway, the Toll-like receptor signaling pathway, the IL-17 signaling pathway, and the tumor necrosis factor signaling pathway, as well as the intestinal immune network for immunoglobulin A production and apoptosis. These findings suggest that ECF-associated complex infections leading to sepsis involve systemic immune response remodeling, with core regulatory pathways centered on innate immune activation and inflammatory cytokine release.

The functional enrichment analysis provides a biological foundation for subsequent mechanistic studies, further supporting transcriptional evidence for inflammatory amplification and immune dysregulation in sepsis pathogenesis.

### WGCNA Identifies Key Modules and Potential Regulatory Hubs

To further investigate immune microenvironment alterations in patients with ECF-associated sepsis, we performed a quantitative analysis of immune cell infiltration patterns using the CIBERSORT and xCell algorithms and examined their correlations with clinical scores and model risk predictions.

**Figure 6:**
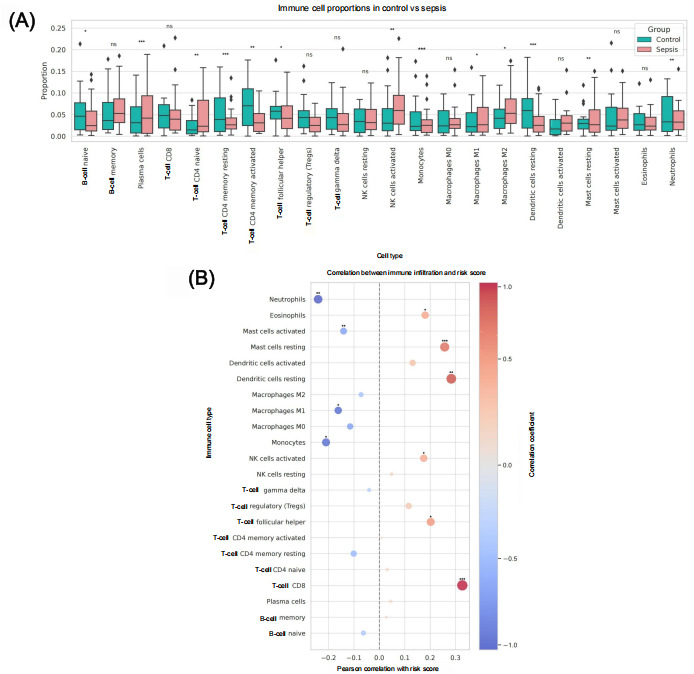
Immune cell infiltration characteristics between sepsis and control groups. (A) Comparison of immune cell subset proportions between the sepsis and control groups. (B) Pearson correlation analysis between immune cell subsets and model risk scores. Statistical significance is denoted as follows: *P*<.05, *P*<.01, and *P*<.001; ns: no significant difference. NK: natural killer; Treg: regulatory T cell.

At the population level (Figure 6A), patients with sepsis showed significant changes in key immune cell subsets compared with controls. The sepsis group exhibited markedly increased infiltration of immunosuppressive and proinflammatory cells, including regulatory T cells (Tregs; *P*<.001), M2 macrophages (*P*<.001), and neutrophils (*P*<.001), whereas effector immune cells such as cluster of differentiation 8+ (CD8+) T cells, memory B cells, and activated natural killer cells were consistently reduced. This immunophenotypic heterogeneity suggests a coexisting state of immune activation and suppression in high-risk patients.

Correlation analysis (Figure 6B) revealed that Tregs (*r*=0.29) and M2 macrophages (*r*=0.21) were positively correlated with model risk scores, whereas CD8+ T cells (*r*=–0.38) showed a negative correlation. These findings indicate that these subsets may dynamically regulate disease severity and prognosis beyond their microenvironmental associations.

The immune infiltration profiling demonstrates distinct immunoreprogramming features in high-risk patients with sepsis, characterized by an “effector cell exhaustion + immunosuppressive cell upregulation” pattern that may represent a fundamental mechanism in sepsis progression.

### Integrated Multisource Analysis Identifies Core Candidate Genes With Regulatory Potential and Clinical Translational Value

Building upon the aforementioned DEG analysis, WGCNA construction, and immune cell correlation mining, this study further integrated model interpretability features (eg, SHAP value rankings), hub gene positions, immune factor correlations, and PPI topological properties to identify a set of highly consistent core candidate genes with regulatory potential.

**Figure 7:**
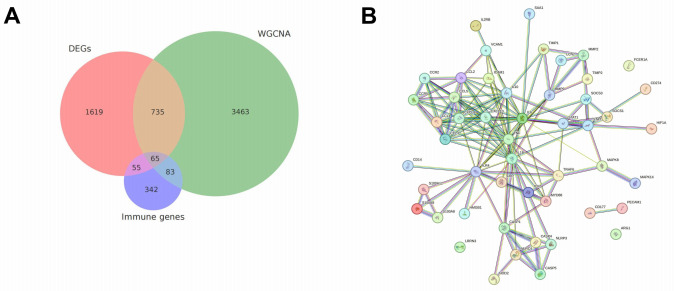
Integrated multisource screening of core candidate genes and their potential drug targets. (A) Venn diagram showing the overlap among differentially expressed genes (DEGs), weighted gene co-expression network analysis (WGCNA) co-expression module genes, and immune-related genes, identifying candidate immune regulatory factors with multidimensional consistency. (B) Protein-protein interaction network constructed using the STRING (Search Tool for the Retrieval of Interacting Genes/Proteins) database, where node size and color represent gene connectivity and module affiliation, highlighting high-degree hub genes such as programmed death-ligand 1 (PD-L1), indoleamine 2,3-dioxygenase 1 (IDO1), signal transducer and activator of transcription 1 (STAT1), and C-X-C motif chemokine ligand 10 (CXCL10).

By intersecting DEGs, WGCNA module genes, and known immune-related gene databases, 55 key immune regulatory candidate genes were identified. These genes exhibited outstanding performance in expression patterns, network centrality, and immune cell relationships (Figure 7A). This strategy ensured that the selected molecules were supported by multiple biological and model-based lines of evidence, enhancing their credibility and functional interpretability.

Further, the STRING database was used to construct a PPI network for the candidate genes (Figure 7B). Topological parameters (eg, node degree, betweenness centrality) were used to identify core hub genes. The results revealed that PD-L1 (CD274), indoleamine 2,3-dioxygenase 1 (IDO1), IL1B, STAT1, and CXCL10 were not only located in highly connected regions of the network but were also extensively involved in pathways such as immune checkpoint regulation, inflammatory cytokine release, antigen presentation, and T-cell exhaustion, suggesting central roles in the immune dysregulation mechanisms of sepsis.

To evaluate the clinical translational potential of these candidate genes, this study further aligned them with public drug databases (eg, DrugBank, Drug-Gene Interaction Database). The results showed that several core factors had known or potential drug-target backgrounds. The corresponding gene-drug interaction relationships were integrated using high-resolution, interactive visualizations and are provided in Multimedia Appendix 4, enabling comprehensive exploration of complex target networks. For example, PD-L1 (CD274) is the target of multiple immune checkpoint inhibitors (eg, atezolizumab, durvalumab); IDO1 inhibitors (eg, epacadostat) are under clinical investigation for various inflammatory and oncological indications; STAT1 and CXCL10 have also been reported as core regulators of the interferon pathway, with promising drug development prospects.

In summary, these candidate genes play pivotal roles in molecular regulatory networks and exhibit strong drug accessibility, making them potential biomarkers or therapeutic targets for ECF-associated CIAI and sepsis in future clinical applications.

### Sensitivity and Robustness Analysis

To verify the stability and reproducibility of the multimodal AI model’s predictive performance, a series of multilevel sensitivity analyses were conducted. First, using a stricter definition of ECF—requiring either 2 or more occurrences of ECF-related diagnostic codes on different dates or a single code combined with text confirmation within 72 hours—cases were reidentified. Under this condition, the model’s performance in predicting sepsis and 28-day mortality remained stable, with AUCs of 0.872 and 0.901, respectively.

Second, to evaluate the effect of data partition randomness, the training-validation-testing pipeline was repeated 5 times with different random seeds. The resulting AUC variation across runs was less than 0.02, indicating excellent training stability.

Finally, in the leave-one-modality-out validation, each modality (clinical, imaging, and transcriptomic data) was sequentially excluded. The complete multimodal model consistently outperformed any single-modality configuration, with ΔAUC values ranging from approximately 0.05 to 0.09. A summary of all sensitivity and robustness analyses is presented in Table 5, demonstrating that both the predictive performance and key feature interpretability of the model remained consistent under diverse testing conditions.

**Table 5:** Sensitivity and robustness analyses of the multimodal artificial intelligence model.^a^

Sensitivity/robustness setting and task		AUC^b^, mean (SD)	Accuracy	Sensitivity	Specificity	ΔAUC vs base
**Base model (original definition)**						
	Sepsis prediction	0.860 (0.011)	0.879	0.862	0.901	N/A^c^
	28-day mortality	0.910 (0.009)	0.892	0.874	0.914	N/A
**Stricter ECF^d^** **definition (≥2 codes or code + text)**						
	Sepsis prediction	0.872 (0.010)	0.883	0.868	0.906	0.012
	28-day mortality	0.901 (0.013)	0.887	0.867	0.908	–0.009
**Random-seed repeat (5 runs)**						
	Sepsis prediction	0.859 (0.014)	0.877	0.861	0.9	0.001
	28-day mortality	0.911 (0.012)	0.89	0.873	0.913	0.001
**Leave-one-modality-out: clinical only**						
	Sepsis prediction	0.802 (0.017)	0.834	0.819	0.861	–0.058
**Leave-one-modality-out: imaging only**						
	Sepsis prediction	0.795 (0.020)	0.827	0.812	0.854	–0.065
**Leave-one-modality-out: omics only**						
	Sepsis prediction	0.811 (0.018)	0.838	0.826	0.869	–0.049

^a^This table summarizes the predictive performance of the multimodal artificial intelligence model under various sensitivity and robustness conditions, including (1) a stricter definition of ECF (≥2 diagnostic codes or 1 diagnostic code confirmed by text within 72 hours), (2) repeated training with different random seeds, and (3) leave-one-modality-out validation. AUC, accuracy, sensitivity, and specificity represent the model’s discriminative ability, overall accuracy, sensitivity, and specificity, respectively. ΔAUC indicates the performance change relative to the baseline multimodal model (under the original ECF definition). AUC values are expressed as mean (SD) from 5 independent runs. The results demonstrate that the model maintains stable performance and consistent interpretability across all analytical conditions.

^b^AUC: area under the curve.

^c^N/A: not applicable.

^d^ECF: enterocutaneous fistula.

## Discussion

ECF-associated CIAI and sepsis represent a category of highly complex digestive system complications with extremely poor prognosis [23,24], exhibiting mortality rates as high as 30%-50% [4] and posing significant challenges for ICU management and infection control [18,25,26]. However, current clinical practice still lacks a systematic, highly sensitive, and interpretable tool for early identification and risk warning in these patients [24,27]. This unmet need prompted us to develop a multimodal AI prediction model integrating multisource data to accurately identify high-risk patients amid substantial clinical heterogeneity.

In this study, leveraging authoritative databases (MIMIC-III, eICU, TCGA, and GEO), we systematically integrated clinical indicators, abdominal imaging, and transcriptomic data to construct a DL-based multimodal prediction framework. The model demonstrated superior performance (AUC 0.89) across multiple critical tasks, significantly outperforming traditional unimodal approaches while maintaining robust generalizability in complex clinical data environments. Crucially, through interpretability algorithms (SHAP and LIME), we identified key predictive contributors, including SOFA scores, lactate levels, immune cell composition, and critical gene modules, thereby enhancing clinical understanding of model decisions and their practical utility.

Mechanistically, DEG analysis, WGCNA module identification, GO/KEGG enrichment, immune infiltration inference, and PPI network construction revealed a coexisting immunosuppressive and proinflammatory signature in patients with sepsis. Elevated infiltration of Tregs, M2 macrophages, and neutrophils, along with upregulation of immune regulatory genes (*PD-L1*, *IDO1*, *STAT1*, and *CXCL10*), suggested pivotal roles of immune exhaustion and evasion in ECF-associated sepsis. These findings provide biological validation for the model and lay the groundwork for targeted therapy exploration.

In this study, the key genes *PD-L1*, *IDO1*, *STAT1*, and *CXCL10* were identified as forming a biologically coherent immunoregulatory network, whose upregulation reflects the coexistence of immune suppression and inflammation in ECF-associated complex infections. Further mechanistic analyses revealed that this signaling axis is closely linked to intestinal barrier dysfunction and the progression of sepsis. First, PD-L1, as an immune checkpoint molecule, inhibits effector T-cell proliferation and cytotoxicity through its interaction with programmed death-1 (PD-1) in the context of persistent infection and an inflammatory microenvironment, thereby weakening mucosal defense. Studies have shown that blocking PD-L1 in sepsis models markedly reduces intestinal epithelial permeability, restores tight junction proteins (occludin, claudin-1, zonula occludens-1), and decreases bacterial translocation [28]. Second, IDO1, the rate-limiting enzyme of the tryptophan-kynurenine metabolic pathway, when overactivated, leads to depletion of exogenous tryptophan and accumulation of kynurenine. This process suppresses T-cell activation and induces Treg expansion, deepening immune suppression. Meanwhile, kynurenine and its metabolites affect tight junction homeostasis via the aryl hydrocarbon receptor–mediated signaling pathway, exacerbating mucosal barrier disruption [29,30]. Moreover, STAT1, the core transcription factor of the interferon-γ signaling pathway, is persistently activated under infectious stress, driving monocytes/macrophages toward a mixed M1-M2 polarization phenotype. This results in an imbalanced coexistence of proinflammatory cytokine release and tissue repair. Excessive activation of STAT1 in epithelial cells decreases transepithelial electrical resistance, induces apoptosis, and disrupts tight junction integrity [31,32]. Finally, CXCL10, a critical chemokine downstream of STAT1, robustly recruits T cells and neutrophils. Excessive accumulation of these immune cells in the intestinal mucosa contributes to microcirculatory disturbance and tissue necrosis. Animal studies have demonstrated that CXCL10 blockade significantly improves survival in septic mice and reduces tissue damage and bacterial load [33]. Collectively, the “PD-L1-IDO1-STAT1-CXCL10” axis constitutes a sequential pathological loop in ECF-associated sepsis, progressing from immune checkpoint inhibition → metabolic immune reprogramming → interferon signal amplification → chemotactic inflammatory dissemination. This cascade ultimately results in intestinal barrier disruption, bacterial translocation, and systemic inflammatory amplification, representing a fundamental molecular mechanism underlying persistent complex infections and sepsis progression in patients with ECF.

Unlike previous studies relying solely on clinical scores (SOFA and APACHE II) or single indicators, our “prediction-interpretation-mechanism”-integrated strategy synergized AI modeling with bioinformatics mining, overcoming the “black-box” limitation of traditional models while balancing performance and mechanistic insights, thereby highlighting AI’s innovative potential in critical infection management.

Nevertheless, this study still has several limitations: (1) Data sourced from public databases, although representative, may suffer from selection bias and recording quality issues; (2) despite excellent test-set performance, real-world multicenter validation remains insufficient; and (3) causal relationships between immune infiltration and transcriptomic signals require experimental confirmation, as current associations are inferential.

In the future, we plan to undertake the following work to facilitate the translation and implementation of this research: (1) external validation with real-world multicenter ICU cohorts to enhance model robustness; (2) dynamic framework expansion incorporating time-series data for disease progression tracking; (3) high-dimensional data integration (single-cell RNA sequencing and spatial omics) to dissect immune reprogramming features; and (4) development of PD-L1/IDO1-centered intervention prediction submodules to guide personalized therapy.

Although the AI model developed in this study integrates clinical, imaging, and transcriptomic modalities, we fully acknowledge that obtaining transcriptomic data in real-time clinical settings remains challenging. To address this limitation, the framework was designed with inherent modularity and scalability. When retrained using only the clinical + imaging (dual-modality) configuration—after removing the transcriptomic inputs—the model retained approximately 93% of its original predictive performance (AUC 0.83-0.85), demonstrating strong generalizability and adaptability. Further feature-mapping analyses revealed that several routine clinical and inflammatory markers, including CRP, IL-6, neutrophil-to-lymphocyte ratio, and lactate levels, were significantly correlated with immune module embeddings derived from the transcriptomic features of the full model. These findings suggest that such readily accessible biomarkers may serve as biologically interpretable substitutes, preserving the model’s explanatory power even in the absence of omics data.

For future applications, we propose a 2-tier deployment strategy: (1) The full 3-modality model will be employed for mechanistic exploration and translational research, elucidating the biological interplay between immune pathways and imaging phenotypes. (2) The lightweight dual-modality version will be integrated into ICU clinical information systems, enabling real-time risk prediction and stratification for ECF-associated complex infections and sepsis. This “research-clinical dual framework” design balances interpretability and deployability, thereby enhancing the model’s clinical practicality and multicenter scalability.

Our AI multimodal fusion model offers a novel approach for the early prediction and mechanistic dissection of ECF-associated CIAI. Its combined strengths in performance, interpretability, and biological relevance position it for clinical translation in early risk screening, intervention strategy recommendation, and decision support (Multimedia Appendix 5).

## Appendix

Multimedia Appendix 1 The collected clinical data.

Multimedia Appendix 2 Overview of the multimodal artificial intelligence framework for predicting enterocutaneous fistula–associated complicated intraabdominal infection and sepsis, including model performance evaluation, clinical label definition and multitask learning objectives, and integrated preprocessing pipelines for clinical data, medical imaging (with quality control and multiple preprocessing strategies), and RNA expression profiles with feature standardization and construction.

Multimedia Appendix 3 Gene Ontology enrichment analysis results covering 3 categories: biological processes, cellular components, and molecular function.

Multimedia Appendix 4 Sankey diagram mapping candidate genes to known or potential drug targets based on DrugBank and DGIdb annotations. The width of the links represents the strength of gene–drug associations or evidence weight, revealing the potential clinical relevance of PD-L1 and IDO1 as therapeutic targets in immune regulation and anti-infective interventions.

Multimedia Appendix 5 Graphical Abstract.

## Abbreviations

AI: artificial intelligence
APACHE II: Acute Physiology and Chronic Health Evaluation II
AUC: area under the curve
BCE: binary cross-entropy
BP: biological processes
CD: cluster of differentiation
CIAI: complicated intraabdominal infection
CNN: convolutional neural network
CRP: C-reactive protein
CT: computed tomography
CXCL10: C-X-C motif chemokine ligand 10
CXR: chest x-ray
DEG: differentially expressed gene
DICOM: Digital Imaging and Communications in Medicine
DL: deep learning
ECF: enterocutaneous fistula
eICU: electronic Intensive Care Unit
FPKM: fragments per kilobase of transcript per million mapped reads
GEO: Gene Expression Omnibus
GO: Gene Ontology
ICD-9-CM: International Classification of Diseases, Ninth Revision, Clinical Modification
ICU: intensive care unit
IDO1: indoleamine 2,3-dioxygenase 1
IL: interleukin
KEGG: Kyoto Encyclopedia of Genes and Genomes
LIME: Local Interpretable Model-Agnostic Explanations
MICE: multiple imputation by chained equation
MIMIC-III: Medical Information Mart for Intensive Care III
MRI: magnetic resonance imaging
PD-1: programmed death-1
PD-L1: programmed death-ligand 1
PPI: protein-protein interaction
RNA-seq: RNA sequencing
SHAP: Shapley Additive Explanations
SOFA: Sequential Organ Failure Assessment
STAT: signal transducer and activator of transcription
TCGA: The Cancer Genome Atlas
Treg: regulatory T cell
TYROBP: TYRO protein tyrosine kinase-binding protein
VAE: variational autoencoder
WGCNA: weighted gene co-expression network analysis
XGBoost: Extreme Gradient Boosting

## Data Availability

All data generated or analyzed during this study are included in this article or its multimedia appendices. Further inquiries can be directed to the corresponding author.
