# Correction: Enterocutaneous Fistula–Associated Sepsis and Mortality: Development and Validation of a Multimodal Artificial Intelligence Prediction Model

**DOI:** 10.2196/104943

**Published:** 2026-07-09

**Authors:** Hui Li, Jing Chen, Peijun Lin, Youmei Pan, Yawen Cao, Wenfeng Xie

**Affiliations:** 1 Department of Emergency Medicine The Sixth Affiliated Hospital of Sun Yat-sen University Guangzhou, Guangdong China; 2 Biomedical Innovation Center The Sixth Affiliated Hospital, Sun Yat-sen University Guangzhou China

In “Enterocutaneous Fistula–Associated Sepsis and Mortality: Development and Validation of a Multimodal Artificial Intelligence Prediction Model” [1], the authors noted one error.

The affiliation has been revised from the following:

1. Department of Emergency Medicine, Biomedical InnovationCenter, The Sixth Affiliated Hospital of Sun Yat-sen University, Sun Yat-sen University, Guangzhou, Guangdong, China

The affiliation has been split into two separate affiliations, as follows:

1. Department of Emergency Medicine, The Sixth Affiliated Hospital of Sun Yat-sen University, Guangzhou, China

2. Biomedical Innovation Center, The Sixth Affiliated Hospital, Sun Yat-sen University, Guangzhou, China

The correction will appear in the online version of the paper on the JMIR Publications website, together with the publication of this correction notice. Because this was made after submission to PubMed, PubMed Central, and other full-text repositories, the corrected article has also been resubmitted to those repositories.
