# Supplementary material for: Enterocutaneous Fistula–Associated Sepsis and Mortality: Development and Validation of a Multimodal Artificial Intelligence Prediction Model
Source: JMIR Med Inform. 2026 Apr 30;14:e79985. doi: 10.2196/79985 (PMC13176812; doi:10.2196/79985)
Supplement: Multimedia Appendix 1 [file medinform_v14i1e79985_app1.pdf]

Patient_ID	Age	Sex	Admission_Type	Fistula_Diagnosis
P0001	69	Male	Urgent	Confirmed
P0002	32	Female	Emergency	Confirmed
P0003	78	Male	Emergency	Suspected
P0004	38	Male	Emergency	Suspected
P0005	41	Female	Emergency	Confirmed
P0006	20	Male	Urgent	Confirmed
P0007	39	Male	Emergency	Confirmed
P0008	70	Male	Elective	Suspected
P0009	19	Male	Elective	Confirmed
P0010	47	Male	Elective	Confirmed
P0011	55	Female	Urgent	Absent
P0012	19	Female	Emergency	Confirmed
P0013	81	Female	Emergency	Absent
P0014	77	Male	Emergency	Confirmed
P0015	38	Male	Urgent	Confirmed
P0016	50	Male	Elective	Suspected
P0017	75	Female	Elective	Suspected
P0018	39	Male	Emergency	Suspected
P0019	66	Male	Elective	Confirmed
P0020	76	Female	Elective	Confirmed
P0021	59	Male	Urgent	Confirmed
P0022	77	Female	Urgent	Suspected
P0023	32	Female	Urgent	Confirmed
P0024	79	Female	Urgent	Absent
P0025	79	Male	Emergency	Confirmed
P0026	64	Male	Urgent	Confirmed
P0027	79	Male	Elective	Suspected
P0028	68	Male	Emergency	Suspected
P0029	72	Male	Elective	Absent
P0030	81	Male	Elective	Confirmed
P0031	20	Male	Elective	Confirmed
P0032	68	Female	Urgent	Confirmed
P0033	24	Male	Urgent	Absent
P0034	38	Female	Emergency	Confirmed
P0035	56	Male	Emergency	Confirmed
P0036	35	Male	Urgent	Confirmed
P0037	21	Male	Elective	Confirmed
P0038	77	Female	Emergency	Confirmed
P0039	31	Female	Urgent	Confirmed
P0040	26	Female	Urgent	Confirmed
P0041	70	Female	Urgent	Suspected
P0042	19	Male	Elective	Absent
P0043	77	Female	Urgent	Confirmed
P0044	61	Male	Urgent	Confirmed
P0045	25	Male	Urgent	Suspected
P0046	64	Female	Urgent	Confirmed
P0047	52	Female	Emergency	Confirmed
P0048	53	Female	Emergency	Confirmed
P0049	67	Female	Urgent	Confirmed
P0050	21	Female	Elective	Suspected

P0051	19	Female	Emergency	Confirmed
P0052	23	Female	Urgent	Suspected
P0053	71	Female	Emergency	Confirmed
P0054	21	Male	Emergency	Confirmed
P0055	71	Female	Elective	Confirmed
P0056	80	Female	Urgent	Confirmed
P0057	35	Male	Urgent	Suspected
P0058	61	Female	Elective	Confirmed
P0059	51	Male	Elective	Confirmed
P0060	79	Male	Urgent	Suspected
P0061	31	Female	Urgent	Confirmed
P0062	65	Male	Elective	Confirmed
P0063	32	Male	Emergency	Confirmed
P0064	79	Male	Emergency	Confirmed
P0065	57	Male	Elective	Suspected
P0066	70	Female	Emergency	Confirmed
P0067	41	Male	Elective	Confirmed
P0068	43	Female	Emergency	Confirmed
P0069	77	Male	Emergency	Suspected
P0070	58	Male	Urgent	Confirmed
P0071	46	Male	Urgent	Confirmed
P0072	32	Male	Emergency	Absent
P0073	62	Female	Emergency	Suspected
P0074	82	Female	Urgent	Suspected
P0075	26	Male	Urgent	Confirmed
P0076	18	Male	Urgent	Confirmed
P0077	25	Female	Elective	Confirmed
P0078	80	Male	Emergency	Confirmed
P0079	28	Male	Urgent	Confirmed
P0080	25	Male	Emergency	Suspected
P0081	52	Female	Elective	Confirmed
P0082	52	Female	Elective	Absent
P0083	50	Female	Urgent	Confirmed
P0084	22	Male	Elective	Suspected
P0085	58	Male	Emergency	Confirmed
P0086	45	Female	Urgent	Confirmed
P0087	24	Female	Emergency	Suspected
P0088	29	Female	Emergency	Confirmed
P0089	51	Female	Elective	Confirmed
P0090	50	Male	Elective	Absent
P0091	65	Female	Elective	Suspected
P0092	40	Male	Elective	Confirmed
P0093	79	Female	Elective	Confirmed
P0094	54	Male	Urgent	Confirmed
P0095	61	Female	Emergency	Suspected
P0096	52	Female	Emergency	Confirmed
P0097	82	Female	Emergency	Confirmed
P0098	64	Female	Emergency	Confirmed
P0099	20	Male	Elective	Confirmed
P0100	18	Female	Emergency	Suspected

CIAI_Presence	Sepsis_Status	SOFA_Score	APACHE_II	ICU_Stay_Days
Yes	No	5.7	12.1	4
No	No	10.6	18.9	22
Yes	Yes	4.2	21.9	2
Yes	No	10.3	17.1	10
Yes	No	6.8	16.4	5
Yes	Yes	9.7	11.6	27
No	No	6.7	12.5	10
Yes	No	4.5	10	1
Yes	No	10.9	5.1	22
Yes	Yes	6.7	14	6
Yes	No	6.5	14.5	13
No	No	8.4	26.9	15
Yes	No	8.8	23.8	4
Yes	No	8.4	18.8	13
Yes	No	1.9	26.4	24
Yes	No	4.1	23	20
Yes	No	4	6	24
Yes	No	3.7	21.3	15
Yes	Yes	3.4	19.6	29
Yes	Yes	4	14.6	8
No	Yes	7	8.9	5
Yes	No	7.5	19	29
Yes	Yes	6	5.6	15
No	No	4.7	15.9	4
No	Yes	9.8	12.9	12
Yes	No	5.7	9.7	13
Yes	No	8.8	21.9	2
Yes	No	4.3	15.8	27
Yes	Yes	7.2	16.9	19
No	No	10	20.5	19
No	No	-1.8	16	4
Yes	Yes	4	18.3	4
Yes	No	7.8	20.1	26
No	Yes	9.6	18.8	11
Yes	Yes	6.6	17.4	27
Yes	No	6.1	24.9	5
Yes	Yes	10.8	22.2	20
Yes	Yes	9.8	15.6	11
No	Yes	3.4	11.5	10
No	Yes	6.3	19.2	8
No	Yes	7.1	17.1	6
Yes	No	11.2	24.5	6
No	No	8.6	20.9	8
Yes	No	5.7	19.8	23
Yes	No	6.7	16.1	15
Yes	No	0.3	11.6	26
No	No	8.9	15	14
No	Yes	7.9	18.4	11
Yes	Yes	9.6	7.4	12
No	Yes	5.6	22.2	26

Yes	Yes	6.4	26.3	13
Yes	No	4.6	6.3	8
Yes	Yes	2.6	9	18
Yes	No	4.2	17.1	25
Yes	No	0.5	13.1	1
Yes	No	8.7	17.3	15
Yes	Yes	-0.5	15.6	8
Yes	Yes	12.1	10.9	28
Yes	No	0.7	22.4	11
Yes	No	10.5	18.3	12
Yes	Yes	3.3	13.2	12
Yes	No	7.7	23.5	4
Yes	Yes	3.7	13.5	16
Yes	Yes	11	9.8	4
Yes	Yes	6.1	16.9	5
Yes	Yes	9.2	17.8	24
No	No	6.2	18.7	5
Yes	No	7.1	19.5	8
Yes	Yes	7.8	18.6	10
No	No	4.4	18.4	17
Yes	No	2.2	13.1	28
Yes	No	2.1	12.6	26
Yes	Yes	6.2	19.7	9
Yes	Yes	4.5	17.2	22
No	Yes	-0.4	20.1	19
Yes	Yes	7.5	7.5	29
Yes	Yes	2.3	13.5	14
Yes	No	2.5	25.5	8
No	Yes	5.9	18.8	9
Yes	Yes	3.3	17.5	22
Yes	Yes	6.4	19.5	11
Yes	Yes	11.9	27	23
No	Yes	4.9	26	1
Yes	Yes	2.8	8.1	14
Yes	Yes	6.5	9.9	5
Yes	Yes	3.7	7.9	21
Yes	Yes	6.1	12.3	26
Yes	No	6.9	16.3	14
Yes	Yes	5.9	17.2	4
Yes	Yes	6.2	8.3	22
Yes	Yes	6.6	6.7	23
No	No	10.8	12.2	25
No	Yes	5.2	20.6	1
Yes	No	6.4	21.5	22
No	No	8.6	24.5	23
Yes	Yes	7.4	15.8	19
Yes	No	4.9	20.1	8
Yes	No	8.7	21	15
Yes	Yes	3.4	13.5	21
Yes	Yes	5.3	17.9	15

28d_Mortality	CRP_Level	WBC_Count	Lactate_Level	Immuno_Phenotype
Alive	153.9	18.6	2.49	Immune imbalance
Alive	32.3	3.6	1.47	Immunosuppression
Deceased	174.4	14.9	7.17	Immunosuppression
Alive	100	8.1	7.67	Immunosuppression
Alive	179.4	18.7	6.97	Immune activation
Alive	161	19.5	6.57	Immune imbalance
Alive	87.9	19.1	5.41	Immune activation
Deceased	9.4	11.1	4.63	Immune activation
Alive	57.4	17.7	1.15	Immunosuppression
Alive	110.6	17.4	3.56	Immune activation
Alive	128.5	8.4	3.3	Immunosuppression
Alive	55.3	17.1	2.45	Immune imbalance
Alive	32.2	3.6	5.93	Immunosuppression
Alive	167.8	13.1	4.22	Immune activation
Alive	197	6.9	1.11	Immune activation
Alive	107.5	5	2.15	Immunosuppression
Alive	38.5	4.3	5.62	Immunosuppression
Alive	58.1	14.8	1.07	Immune activation
Deceased	8.6	8.8	6.88	Immune imbalance
Alive	183.3	15.3	4.21	Immune imbalance
Alive	28	4.1	4.1	Immunosuppression
Alive	117.4	8.4	4.94	Immune imbalance
Alive	58.4	12.2	6.69	Immune activation
Alive	113.1	16.4	3.11	Immunosuppression
Alive	132	8.4	5.59	Immune activation
Deceased	166.8	13.6	4.74	Immune activation
Alive	45.3	18.1	2.5	Immune imbalance
Alive	7.1	13.5	7.09	Immunosuppression
Alive	31.7	7	6.48	Immunosuppression
Deceased	180.5	3.4	5.44	Immune imbalance
Alive	175.4	17.8	6.88	Immune activation
Alive	121.5	3.4	7	Immune activation
Alive	122.1	17.9	5.81	Immune activation
Alive	134.7	12	6.78	Immune activation
Alive	39.2	19	5.73	Immunosuppression
Deceased	183.3	16.6	5.6	Immunosuppression
Alive	86.7	20	5.14	Immune imbalance
Alive	79.7	9	6.15	Immune imbalance
Alive	106.2	16	1.69	Immunosuppression
Alive	14.2	9.8	7.11	Immunosuppression
Alive	37.4	11.2	7.04	Immune activation
Alive	148.9	13.7	0.72	Immune activation
Deceased	21.1	17.9	6.69	Immune imbalance
Alive	122.6	19.7	1.47	Immunosuppression
Alive	52.8	16.1	3.01	Immune imbalance
Alive	80.9	10.1	6.08	Immunosuppression
Alive	61.3	10.2	1.71	Immune activation
Alive	74.4	15.5	6.63	Immune imbalance
Alive	145.2	7.1	6.74	Immune imbalance
Alive	62.9	4.9	4.31	Immunosuppression

Deceased	115.4	9	0.55	Immune activation
Deceased	97.8	7.9	2.65	Immune imbalance
Alive	134.4	8	5.13	Immune activation
Alive	187.7	7	7.86	Immune imbalance
Alive	147.9	3.7	5.24	Immune activation
Alive	46.9	3.3	2.45	Immunosuppression
Alive	11.1	19.8	5.26	Immune imbalance
Alive	56.1	10.3	4.55	Immune imbalance
Alive	121	9.5	6.35	Immune activation
Alive	15	14.6	1.3	Immune activation
Alive	101.8	6.7	6.21	Immune imbalance
Alive	121.4	19.1	4.56	Immunosuppression
Alive	70.2	16.4	7.72	Immune activation
Alive	155.3	4.5	3.06	Immune activation
Alive	25.8	10.1	5.24	Immune activation
Alive	19.7	17.9	7.49	Immune imbalance
Deceased	147	19.1	1.27	Immune imbalance
Alive	101.6	10.9	7.53	Immune imbalance
Alive	139.2	13.4	5.66	Immune imbalance
Alive	89.8	5.8	1.01	Immunosuppression
Alive	53	19.8	2.76	Immunosuppression
Alive	164.7	6.9	5.81	Immune imbalance
Alive	160.9	19	1.01	Immunosuppression
Deceased	140.5	14	4.87	Immune activation
Deceased	58.1	13.3	3.09	Immune activation
Alive	120.1	11.7	5.16	Immune activation
Alive	75.4	6.9	0.84	Immune imbalance
Alive	22.9	6	7.04	Immune activation
Alive	183.9	6.7	7.8	Immunosuppression
Deceased	31.7	6.2	7.77	Immune imbalance
Alive	190.3	16.3	6.12	Immunosuppression
Alive	92	9	1.48	Immune activation
Alive	41.1	4	6.19	Immune activation
Alive	110.7	19.5	0.68	Immune activation
Alive	175.2	18	0.67	Immune imbalance
Alive	147.8	18.8	2.93	Immunosuppression
Alive	162.3	19.9	4.16	Immune imbalance
Deceased	133.5	6	6.28	Immune imbalance
Deceased	140	9.7	5.62	Immune activation
Alive	170.6	15.9	3.84	Immune imbalance
Alive	53.7	14.8	2.55	Immune imbalance
Alive	100.4	5.6	7.98	Immune activation
Alive	48.1	16.9	3.7	Immune imbalance
Alive	197.6	6.8	3.89	Immune activation
Alive	189.1	6.8	1.73	Immune activation
Alive	12.7	12.1	6.46	Immune activation
Alive	142.6	13.1	5.7	Immune activation
Alive	185.4	12.9	2.16	Immune imbalance
Alive	40.2	4.6	1.12	Immune activation
Deceased	115.7	17.9	5.6	Immune activation
