# Supplementary material for: Enterocutaneous Fistula–Associated Sepsis and Mortality: Development and Validation of a Multimodal Artificial Intelligence Prediction Model
Source: JMIR Med Inform. 2026 Apr 30;14:e79985. doi: 10.2196/79985 (PMC13176812; doi:10.2196/79985)
Supplement: Multimedia Appendix 2 [file medinform_v14i1e79985_app2.docx]

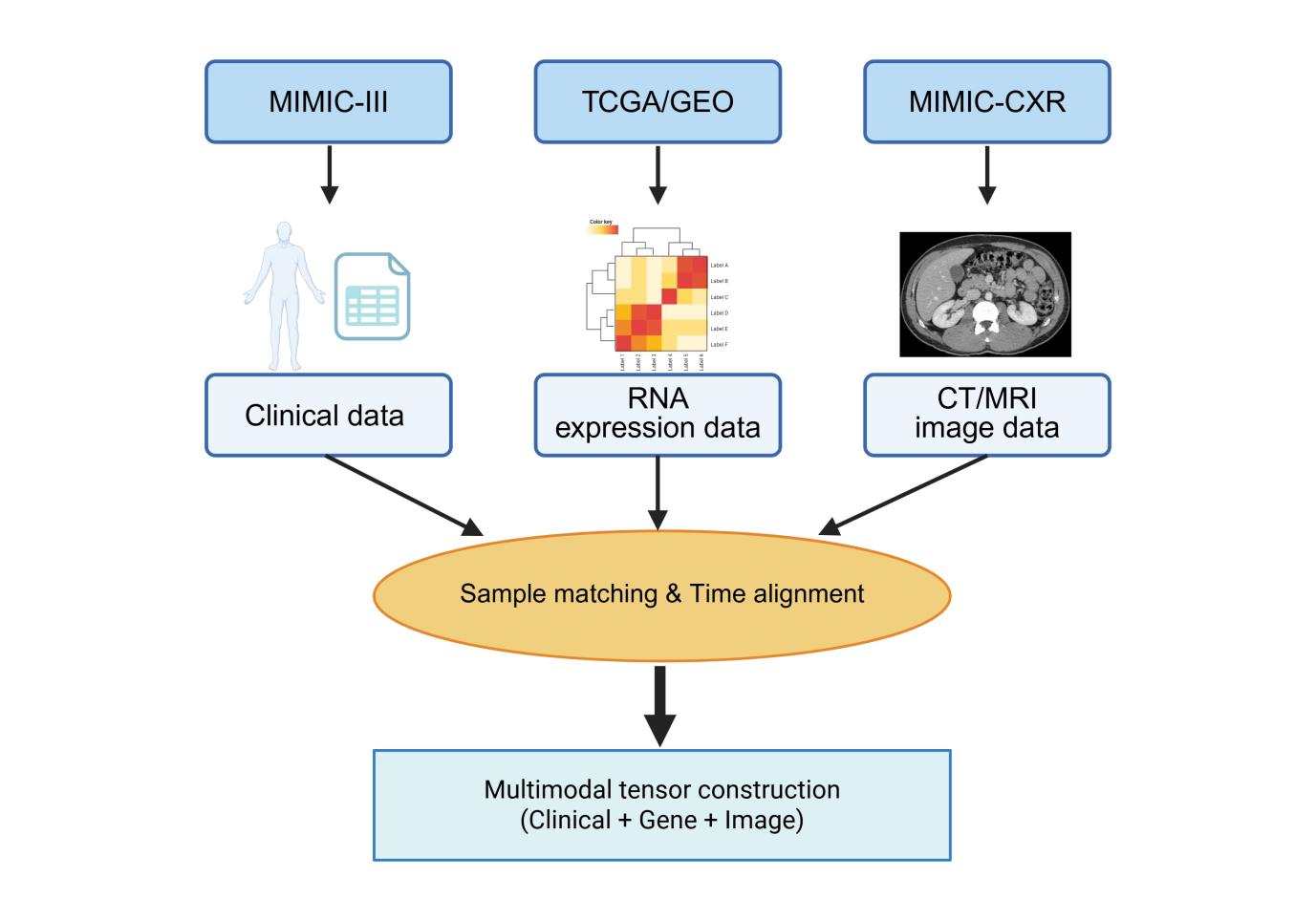


**Figure S1. Performance evaluation of multimodal AI model for predicting ECF-associated CIAI and sepsis.**


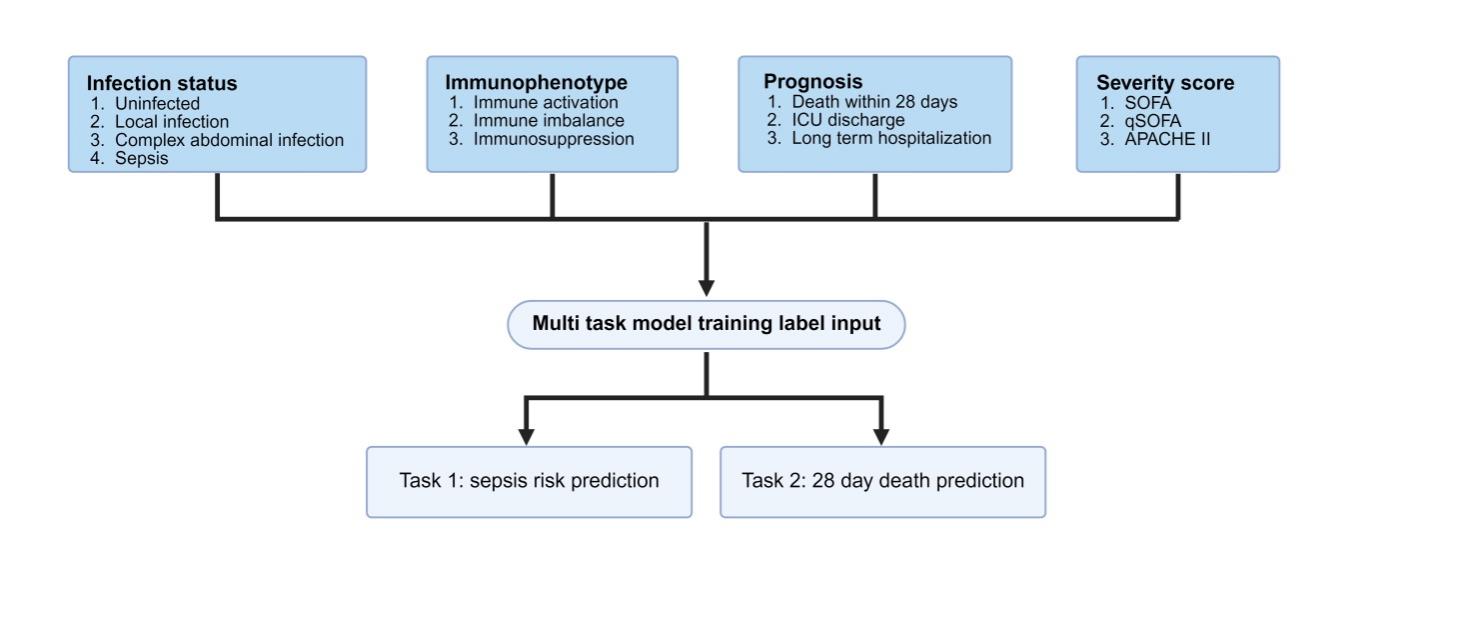


**Figure S2. Clinical label definition and model training objectives under multitask learning framework.**


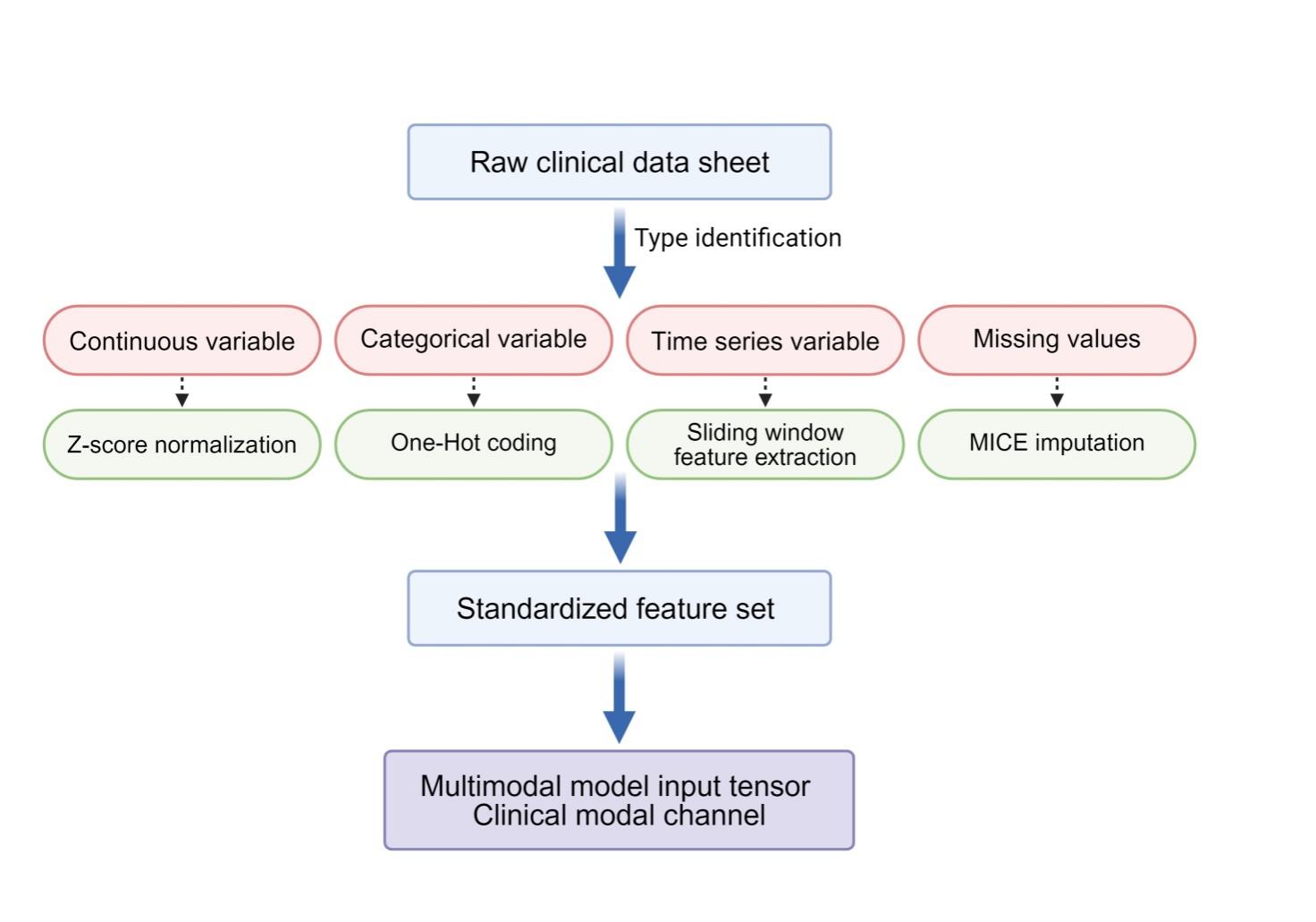


**Figure S3. Clinical data preprocessing and feature standardization workflow.**


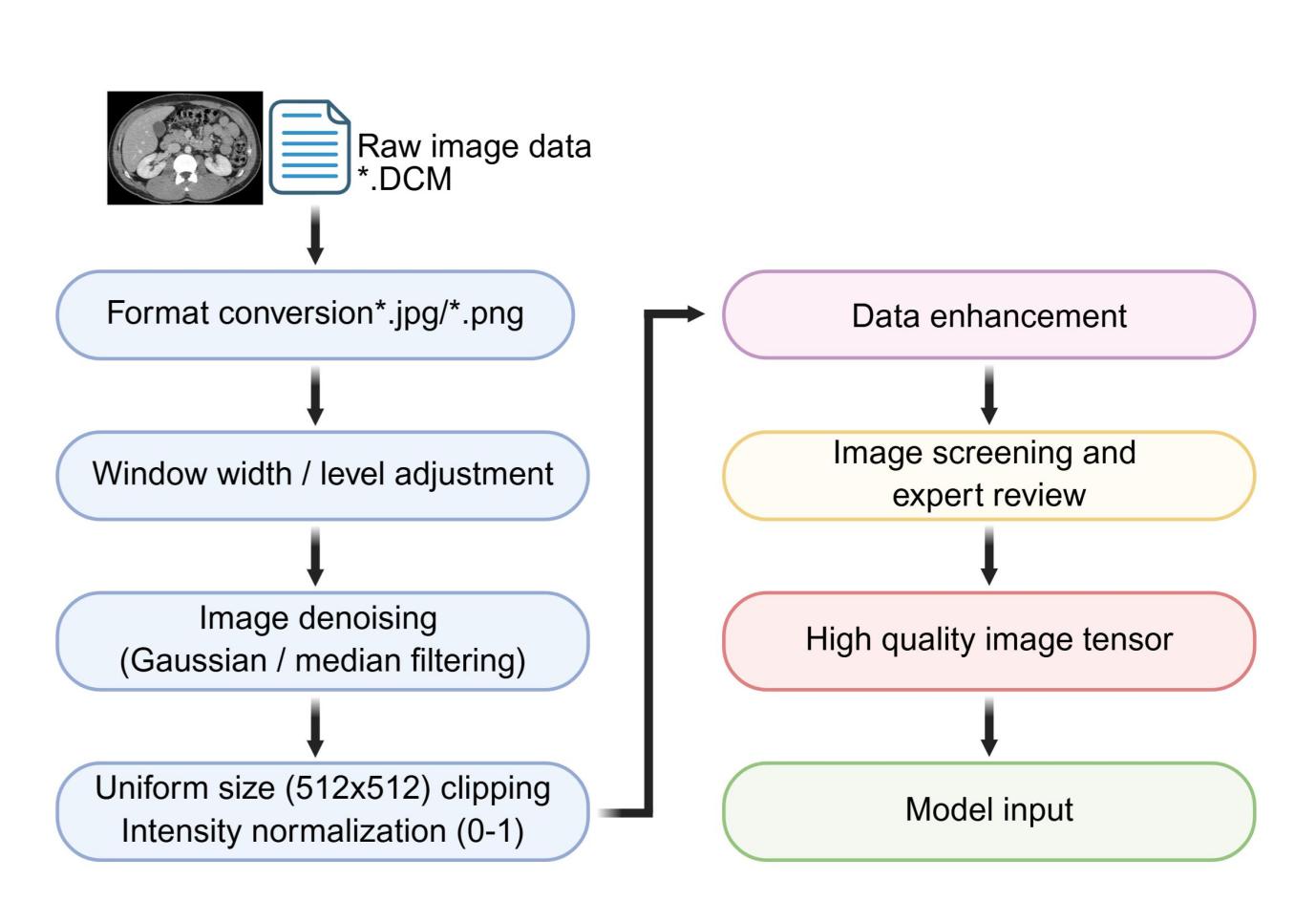


**Figure S4. Medical imaging preprocessing and quality control workflow.**


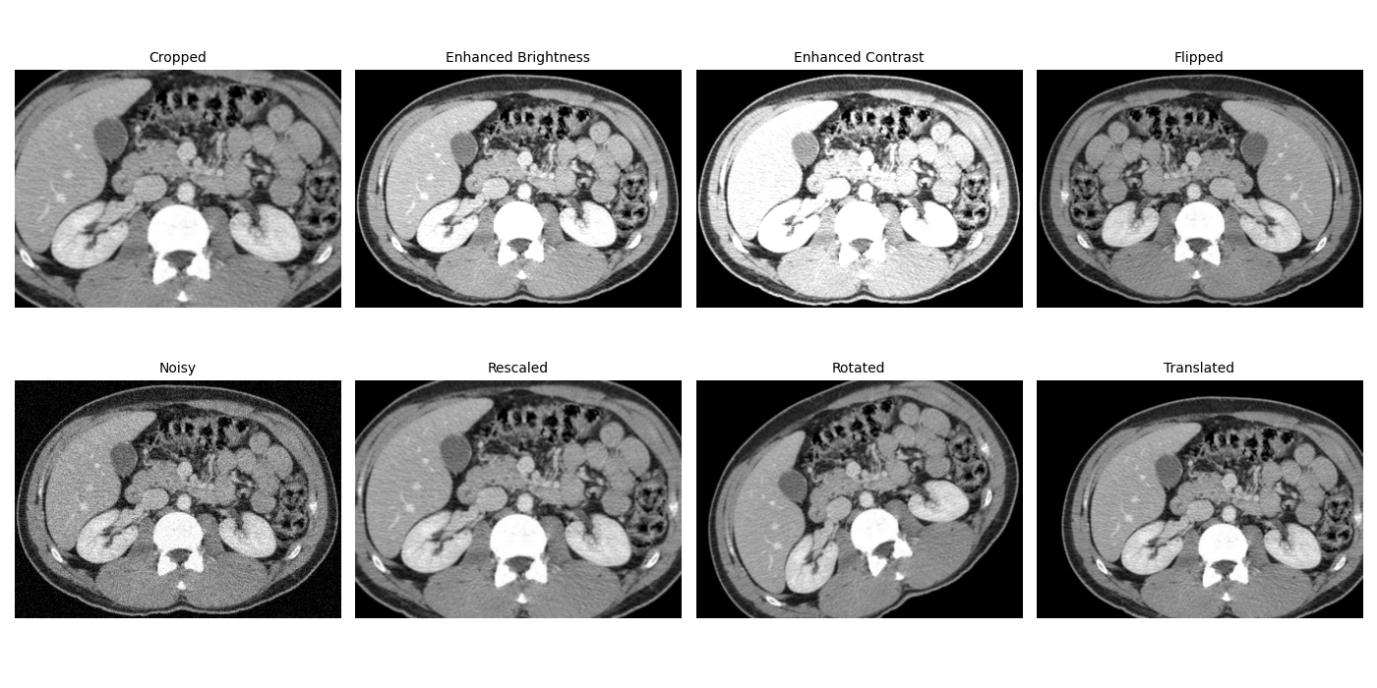


**Figure S5. Eight preprocessing methods for imaging data.**


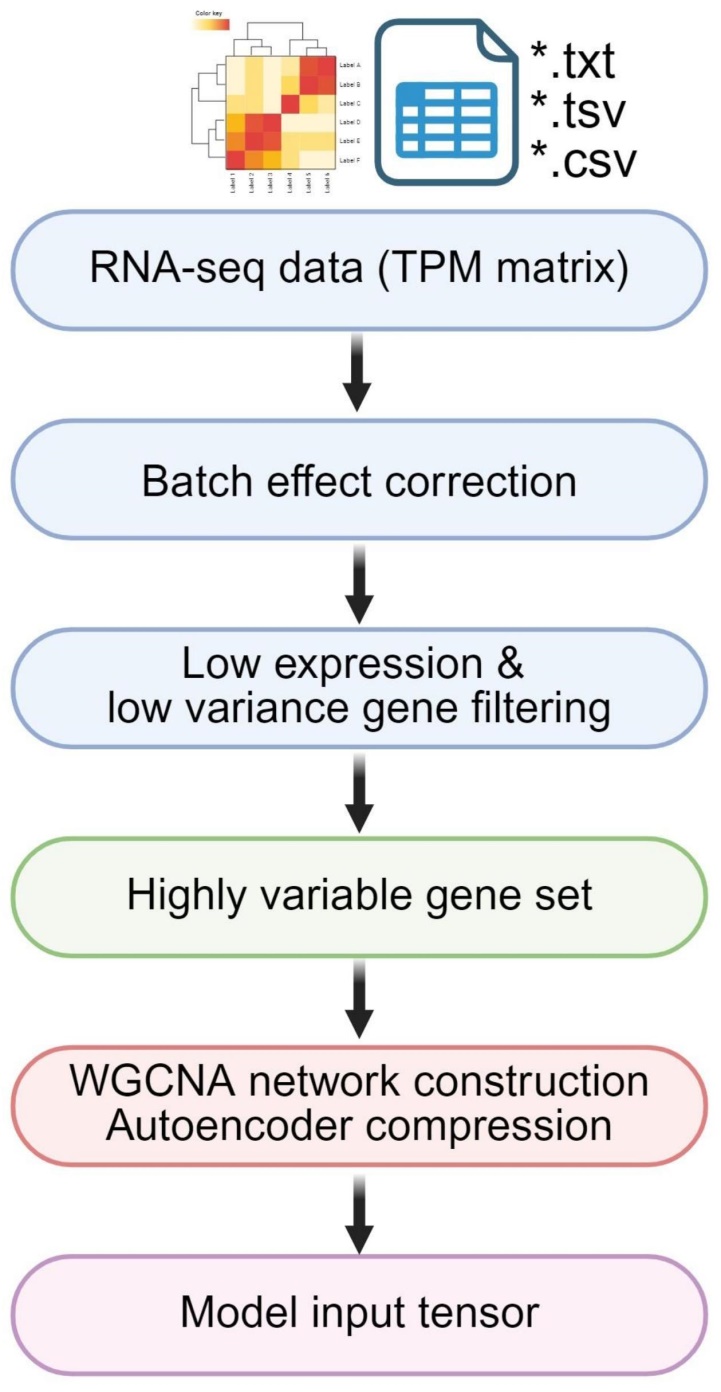


**Figure S6. RNA expression profile standardization and feature construction workflow.**


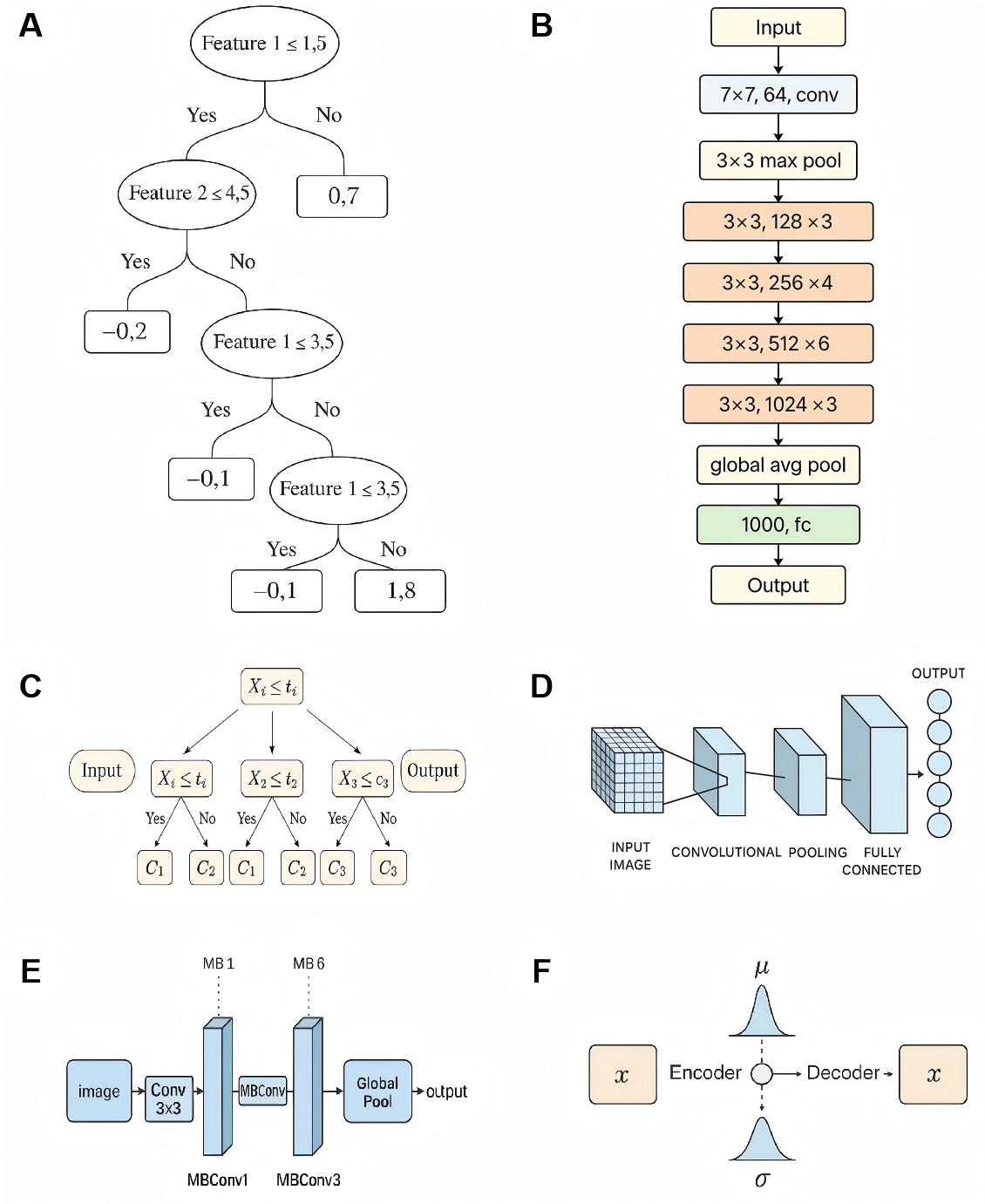


**Figure S7. RNA expression profile standardization and feature construction workflow.**
