# Supplementary figures and images for: Enterocutaneous Fistula–Associated Sepsis and Mortality: Development and Validation of a Multimodal Artificial Intelligence Prediction Model
Source: JMIR Med Inform. 2026 Apr 30;14:e79985. doi: 10.2196/79985 (PMC13176812; doi:10.2196/79985)

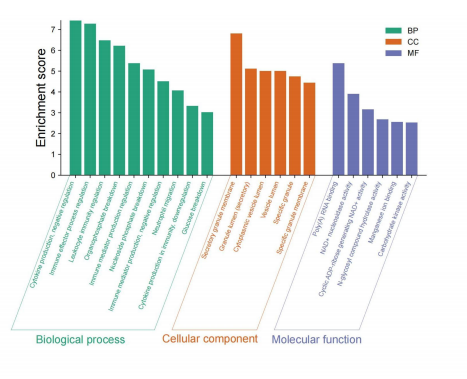

Supplement: Multimedia Appendix 3 [file medinform_v14i1e79985_app3.png]

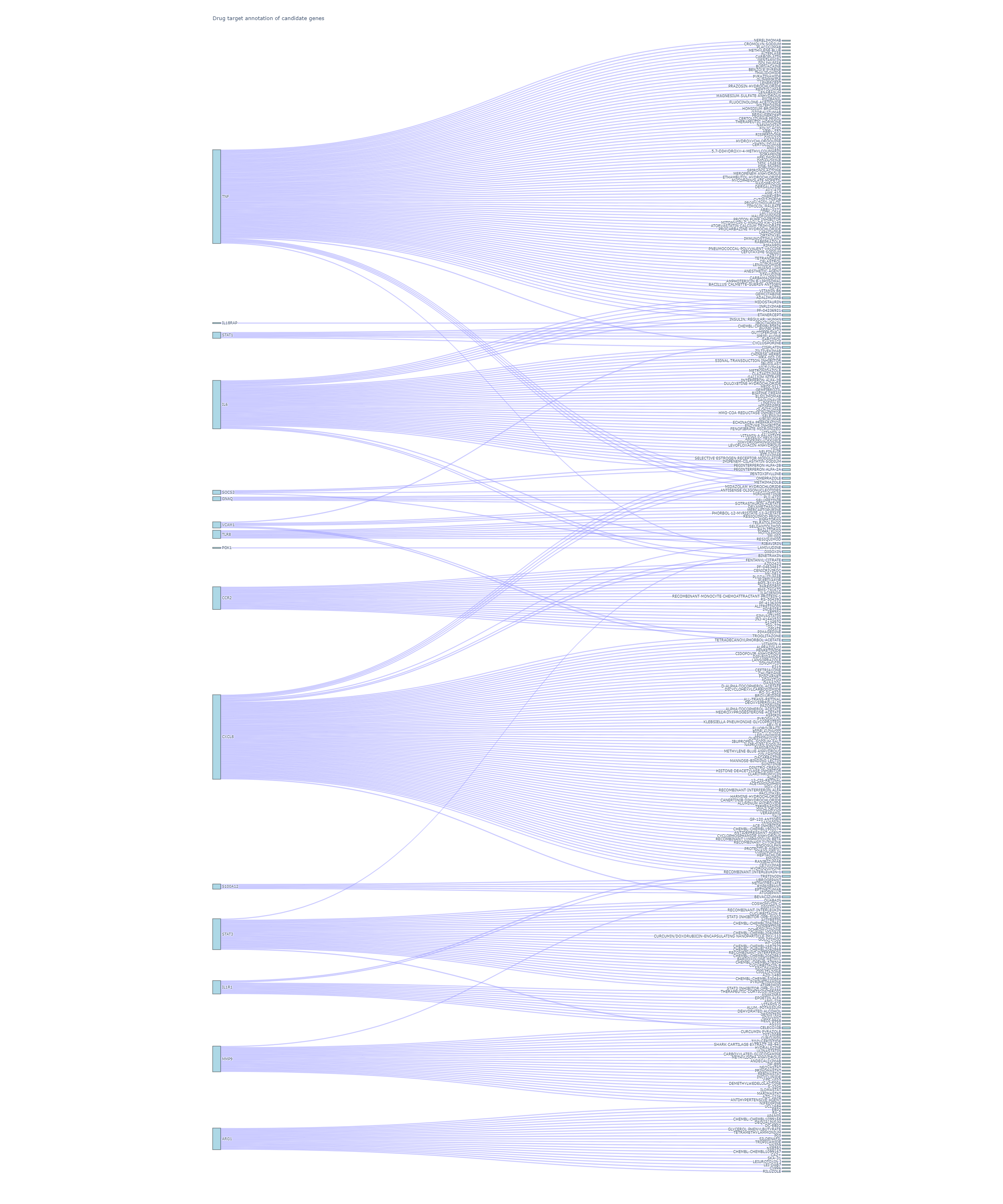

Supplement: Multimedia Appendix 4 [file medinform_v14i1e79985_app4.png]

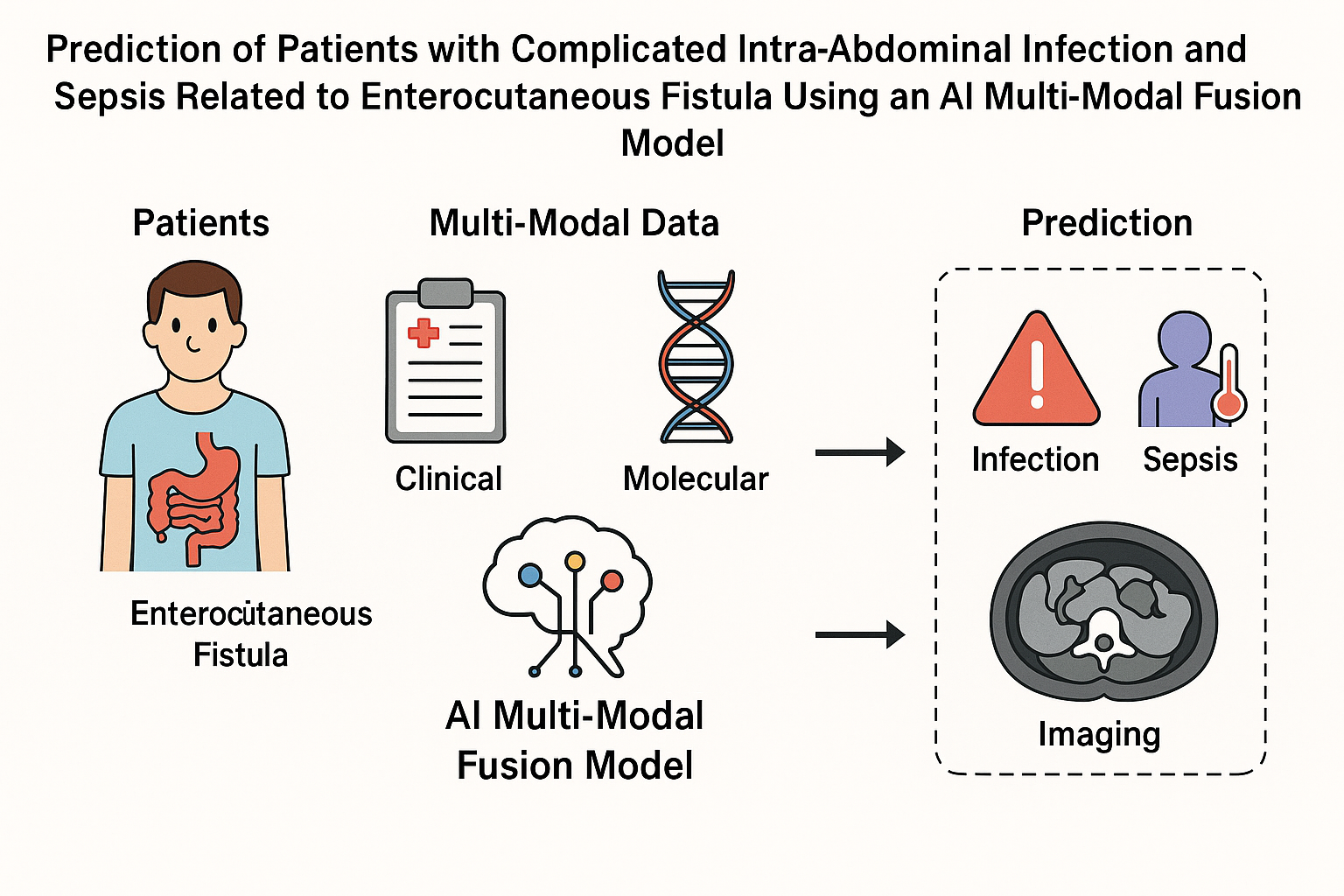

Supplement: Multimedia Appendix 5 [file medinform_v14i1e79985_app5.png]
